# Molecular and phenotypic insights into sulfur’s role in enhancing tomato plant growth, stress tolerance, and productivity

**DOI:** 10.1038/s41598-025-17645-3

**Published:** 2025-09-25

**Authors:** Junwoo Lee, Jung Heo, Eun Song Lee, Hye-yeong Kang, Smita Mirsyad Warsadiharja, Keunhwa Kim, Yousun Chae, Aditya Nurmalita Pervitasari, Ryza A. Priatama, Young Koung Lee, Il Ho Kim, Soon Ju Park

**Affiliations:** 1 Division of Applied Life Science and Plant Molecular Biology and Biotechnology Research Center (PMBBRC), Gyeongsang National University, Jinju, 52828 Republic of Korea; 2 Department of Plant Science and Technology, Chung-Ang University, Anseong, 17546 Republic of Korea; 3 Institute of Plasma Technology, Korea Institute of Fusion Energy, 37 Dongjangsan-ro, Gunsan-si, 54004 Jeollabuk-do Republic of Korea; 4 Nara Bio Research & Development Center Co., Ltd., Gunsan, 54006 Jeollabuk-do Korea

**Keywords:** Sulfur fertilization, Transcriptome, Translation capacity, Stress, Resistance, Productivity, Plant sciences, Plant molecular biology, Plant physiology, Plant reproduction

## Abstract

Sulfur is an essential nutrient for plant growth and defense, participating in biochemical processes like gene expression, translation, and redox reactions. While its role in sulfur deficiency has been well studied, the biological responses to sulfur fertilization remain less explored. Here, we investigated these responses by analyzing gene expression in tomato at both hourly and daily intervals following sulfur fertilization. Gene ontology (GO) analysis of differentially expressed genes (DEGs) in response to sulfur fertilization revealed increased expression of genes related to water and wounding responses, as well as cell wall biosynthesis within a day. Weighted gene co-expression network analysis (WGCNA) identified 14 modules linked to time-dependent gene expression changes after sulfur treatment. MAPK and hormone signaling genes peaked within one hour, followed by enhanced molecular expression pathways involved in mRNA transport, ribosome biogenesis, and amino acid biosynthesis. Modules that peaked more than 1 day after sulfur treatment were enriched in processes related to glutathione metabolism, cell division, and auxin and steroid biosynthesis, which are likely to contribute to sustained growth and stress resistance. These findings were validated by increased drought and TYLCV resistance, as well as improved yield in sulfur-fertilized tomato plants, highlighting sulfur fertilization as a strategy for improving agricultural productivity.

**Supplementary Information:**

The online version contains supplementary material available at 10.1038/s41598-025-17645-3.

## Introduction

Sulfur is an essential nutrient that plays a pivotal role in plant physiology and development^1–3^. As a constituent of vital amino acids such as methionine and cysteine, sulfur is integral to protein synthesis and redox control in plants^4^. Additionally, it contributes to the formation of important molecules like glutathione, which serves a key function in protecting plants from oxidative stress by scavenging reactive oxygen species (ROS)^5,6^. Sulfur’s involvement extends to the synthesis of vitamins, coenzymes, and secondary metabolites that are crucial for plant metabolic functions and growth^3,7^. Moreover, sulfur is especially important in the formation of disulfide bonds in proteins, which are critical for maintaining protein structure and function under various environmental stresses^8^. Furthermore, sulfur-containing compounds, such as Fe-S cluster proteins, are fundamental in processes like photosynthesis and energy metabolism^9^.

The demand for sulfur increases significantly during key growth phases, such as seed development and vegetative growth, as these processes require a higher supply of sulfur-containing metabolites^10^. In recent years, the combination of modern agricultural practices and environmental factors has reduced atmospheric sulfur inputs, leading to lower sulfur availability in soils due to cleaner air policies, the increased use of sulfur-free fertilizers, and the reduced application of sulfur-containing pesticides and fungicides^11,12^. This deficiency presents substantial challenges in maintaining crop yields. As a result, crops suffer from reduced vigor, decreased yield, and diminished resistance to environmental stress^13^.

At the molecular level, sulfur scarcity triggers complex biological responses in plants, influencing the regulation of gene expression, the synthesis of proteins, and key metabolic pathways^14^. It also affects epigenetic regulation, such as histone modifications and DNA methylation, through sulfur-derived metabolites like S-adenosylmethionine (SAM)^15^. Specifically, sulfur-deficient plants upregulate sulfur transporter genes (*SULTR* family) and enzymes involved in sulfate assimilation, such as ATP sulfurylase and APS reductase, to enhance sulfur uptake and metabolism^16,17^. This deficiency disrupts protein synthesis, particularly in crops like wheat, where grain quality is affected, while simultaneously triggering oxidative stress through reduced glutathione levels, which leads to increased ROS and prompts plants to compensate by upregulating antioxidant-related genes^14,18,19^. Additionally, sulfur deficiency reduces chlorophyll content, impairing photosynthesis^20^. Crosstalk with nitrogen metabolism pathways further complicates the response to sulfur scarcity^18^. However, sulfur fertilization restores gene expression, boosting protein synthesis, photosynthesis, and stress resistance^21^.

Although extensive research has demonstrated the benefits of sulfur fertilizers in enhancing plant health, there remains a significant gap in understanding the molecular mechanisms through which sulfur impacts plant biology at the gene expression level^1,22^. Studies on the biological effects of sulfur application typically focus on macroscopic traits such as growth rate and yield, but they lack comprehensive insights into the underlying molecular processes^1^. In particular, the transcriptional and post-transcriptional changes induced by sulfur treatments in plants are poorly characterized. Therefore, further research is needed to investigate the molecular responses triggered by sulfur application, particularly in crops like tomato, where gene expression analysis could offer new insights into sulfur’s effects on growth, stress resilience, and overall productivity.

## Results

### Sulfur treatment to determine the optimum dose of sulfur to promote plant growth

**Fig. 1:**
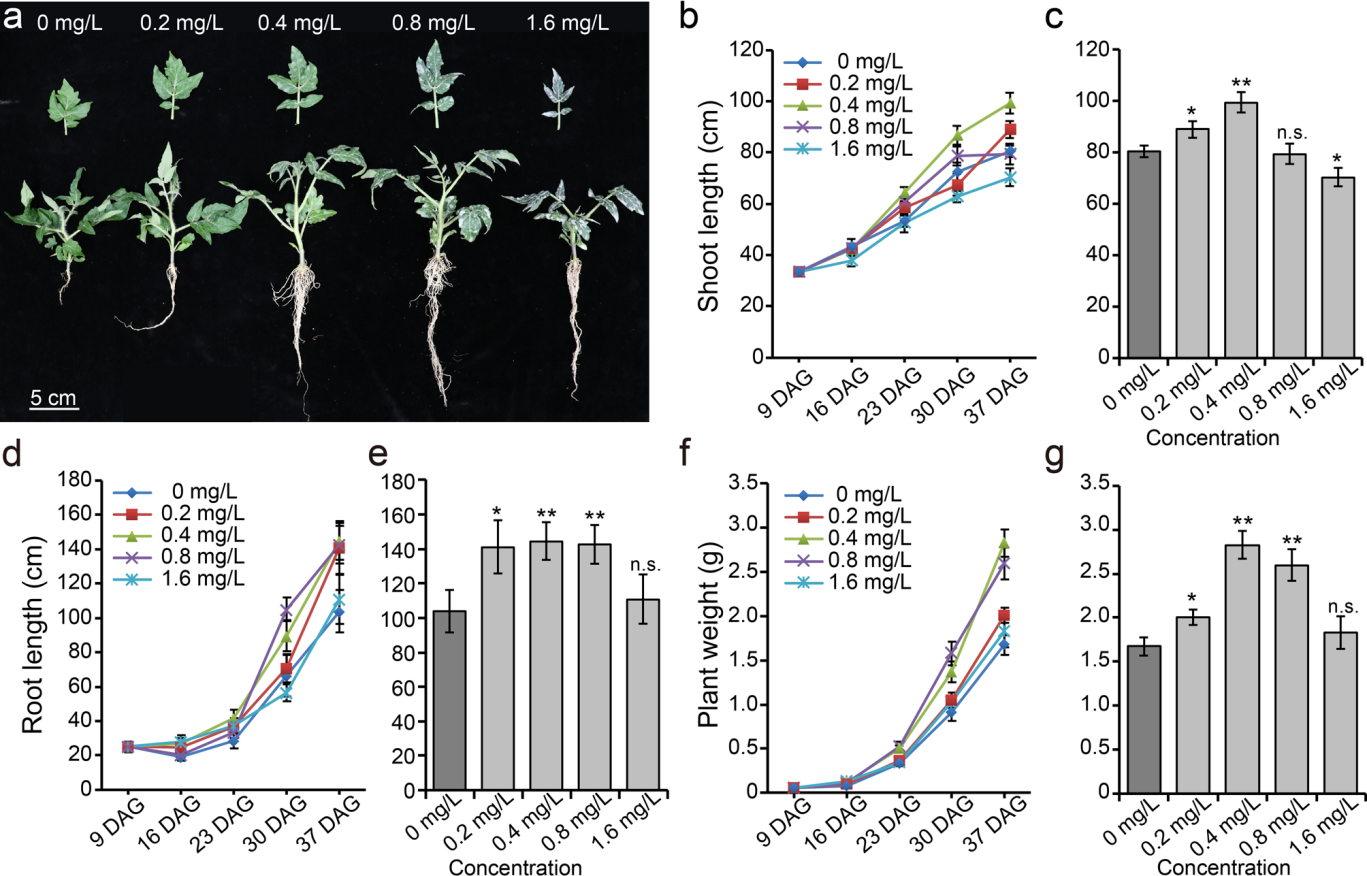
Plant growth responses to elemental sulfur treatments over 4 weeks. (**a**) Representative images of tomato plants grown under five sulfur concentration treatments (0, 0.2, 0.4, 0.8, and 1.6 mg/L). Scale bar: 5 cm. (**b**, **d**, **f**) Line graphs showing shoot length (b), root length (d), and plant weight (f) measured at different time points (9, 16, 23, 30, and 37 DAG) under varying sulfur concentrations. Sulfur treatments were applied weekly for four weeks, starting at 9 DAG. (**c**, **e**, **g**) Bar graphs displaying shoot growth (c), root growth (e), and plant weight (g) of 37 DAG plants treated with sulfur. Asterisks indicate statistically significant differences compared to the control (0 mg/L) (*p < 0.05, **p < 0.01, n.s. = not significant), based on a two-way Student’s *t*-test.

For tomato grown in soil, pure elemental sulfur treatment has the advantage of providing a continuous supply of sulfur to the plants. To select the most effective concentration of elemental sulfur for plant growth, we sprayed five different concentrations of elemental sulfur (0, 0.2, 0.4, 0.8, and 1.6 mg/L) onto the whole plant and soil of nine-day-old plants. Four times, sprays of sulfur were applied at seven-day intervals to measure plant growth. Overall, sulfur treatment had a positive effect on stem, root, and leaf growth, with significant growth promotion observed at three weeks after sulfur treatment at concentrations of 0.2, 0.4, and 0.8 mg/L (Fig. 1). However, plants at 1.6 mg/L were similar in size to control plants treated water (0 mg/L), but the leaves and stems of the plants were dark green compared to the control (Fig. 1a). The concentration that enhanced the most stem growth was 0.4 mg/L in the third and fourth week, and the 0.2 mg/L treatment showed the most growth stimulation in the fourth week, which is thought to be due to the accumulated weekly elemental sulfur concentrations (Fig. 1b and c). The slowdown of 0.8 mg/L treatment in stem growth after the third week is supported by sulfur accumulations, as 1.6 mg/L sulfur treatment had no significant effect on plant stem growth (Fig. 1b and c). Interestingly, root growth of all 0.2, 0.4, and 0.8 mg/L sulfur-treated plants showed a significant increase in the fourth week, and the decrease in root growth rate in 0.8 mg/L sulfur-treated plants after three weeks was mild compared to the changes in stem growth (Fig. 1d and e). To comprehensively assess these growth differences, the plant mass increase following sulfur treatment was measured, with the highest plant weight in the 0.4 and 0.8 mg/L sulfur treatments, with the 0.4 mg/L treatment showing an increase in plant weight from four weeks due to the cumulative sulfur treatment dose (Fig. 1f and g).

### Time-course transcriptome-profiling after sulfur treatment

To investigate the response of tomato plants to sulfur fertilization, 21-day-old seedlings were treated with elemental sulfur at a concentration of 0.4 mg/L. Whole plants except roots were collected at hourly intervals (0, 1, 3, 6, 24 h) and daily intervals (0, 1, 2, 3 days) in three biological replicates for RNA sequencing (Supplementary Fig. S1). On average, 51,261,030 clean reads per sample were mapped to the full-length tomato transcripts (ITAG 4.1), and gene expression was quantified in Transcripts Per Million (TPM). A total of 18,535 genes were expressed with at least 1 TPM value (Supplementary Table S1). To assess the variation in expression between replicates and samples of different time points, we conducted Principal Component Analysis (PCA) and a heatmap using correlation analysis. Differences in expression between replicates were minimal, indicating minor bias from sampling, with over 70% of the variance across samples explained by PC1 and PC2 (Supplementary Fig. S1b). Notably, gene expression profiles from hourly intervals showed significantly greater variations compared to those from daily intervals (Supplementary Fig. S1c). Additionally, we conducted measurements of sulfur content in root and shoot tissues (stem and leaves) to evaluate the absorption and distribution of sulfur following elemental sulfur application to the soil. Sulfur content in the roots showed a continuous increase over time, while only a slight increase was observed in the shoot tissues. (Supplementary Fig. S2). The results revealed that elemental sulfur is efficiently absorbed through the roots, while sulfur levels in the shoot may be actively regulated by the plant.

### Time-dependent expression changes after sulfur treatment (DEGs)

To understand how gene expression changes over time after sulfur treatment, DESeq2 analysis was conducted using the criteria log2-fold change ≥ ± 1, FDR < 0.1, and TPM ≥ 1. This analysis identified 2,754 differentially expressed genes (DEGs) for hourly changes (Supplementary Table S2) and 268 DEGs for daily changes (Supplementary Table S3). These DEGs were then grouped into 10 clusters for hourly intervals and 8 clusters for daily intervals using Mfuzz K-means clustering. For each cluster (C), the peak gene expression following sulfur treatment was determined. Clusters with high correlations were further examined using GO term enrichment to explore their biological functions (Supplementary Figs. S3 and S4).

In the early (hourly) response to sulfur treatment, sulfur transporter gene expression (Supplementary Fig. S3b) decreased significantly during the first 1 and 3 h but recovered by the 24-hour mark. GO term analysis using DEGs of the 10 clusters identified two major groups among the clusters: the ‘reduced early response’ group (C1-C4), which includes genes involved in sulfur and transmembrane transport, and the ‘increased early response’ group (C5-C10), comprising genes related to transcription factor activity, calmodulin binding, and membrane and cell wall synthesis. These changes suggest an initial adaptation mechanism to manage excess sulfur (Supplementary Fig. S3).

In the long-term (daily) response, GO term analysis of DEGs from eight clusters also revealed two major groups: the ‘reduced response’ group (C1-C3) and the ‘increased response’ group (C4-C8). The ‘increased response’ group showed enrichment in genes linked to peptidase inhibitor activity, water and wounding responses, and cell wall and chitin catabolism. These results indicate that sulfur treatment over time enhances processes associated with wound repair, pathogen resistance, and stress tolerance, such as drought resilience (Supplementary Fig. S4).

### Co-expression gene group (module) identification after sulfur treatment

**Fig. 2:**
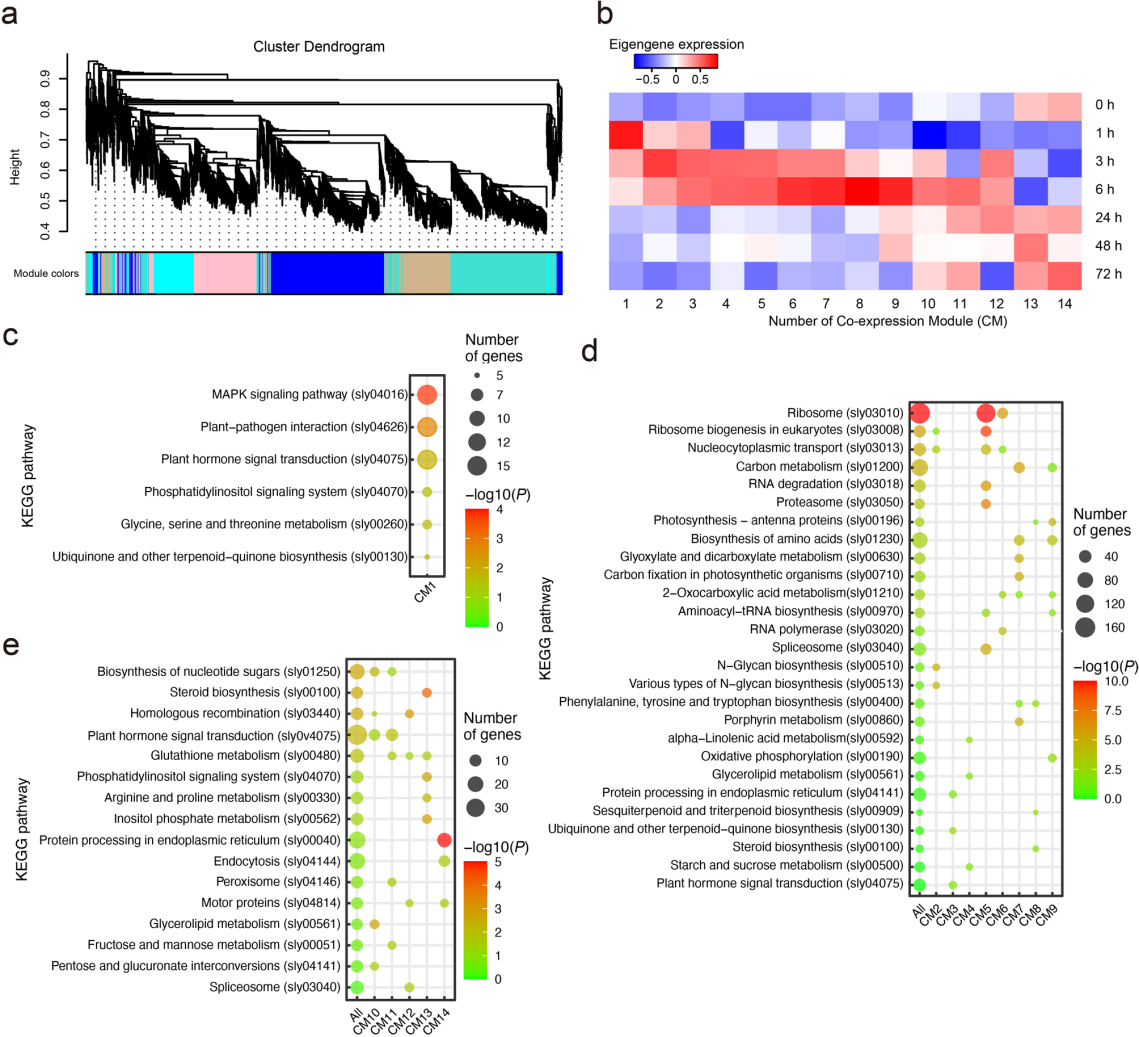
WGCNA module identification and functional analysis over time after sulfur treatment. (**a**) Cluster dendrogram illustrating the identification of co-expression modules (CMs) using WGCNA. Modules are represented by distinct colors, grouping genes with similar expression patterns. (**b**) Heatmap of eigengene expression values for each module across time points after sulfur treatment. Red and blue indicate the strength of eigengene expression, respectively. CMs are ordered based on the peak time of their expression dynamics. (**c**) KEGG pathway enrichment analysis for CM1, highlighting significantly enriched pathways. (**d**) KEGG pathway enrichment analysis of CM2 to CM9, representing the early response to sulfur treatment. (**e**) KEGG pathway enrichment for CM10 to CM14, representing the late response to sulfur treatment. Dot size indicates the number of genes, and color represents the significance level (-log10(P)). ‘All’ indicate to the entire set of the group’s CMs.

DEG analysis, while informative, may have limitations due to the relatively small number of genes analyzed, which may not fully capture the expression changes in the plant system. To analyze co-expression networks with correlations of expression patterns in plant systems following sulfur treatment, we selected 12,062 genes showing a baseline expression (with an average TPM of 3 or more) and having log2(TPM + 1) values within the top 75% median absolute deviation (MAD) and then grouped them into 14 co-expressed gene clusters (modules) through the WGCNA algorithm (WGCNA-R) (Supplementary Fig. S5, Supplementary Table S4). The modules were confirmed to have no outliers and proximity between samples. Through analysis of scale independence and mean connectivity, the optimal soft threshold parameter was determined to be 15 (Fig. 2a, Supplementary Fig. S5).

To better understand the overall gene expression patterns, a heatmap of gene expression values for each of the 14 modules over time following sulfur treatment was generated and ordered (Fig. 2b, Supplementary Fig. S5c). Co-expression Module 1 (CM1) represents a group of genes that exhibit changes in expression within 1 h of sulfur treatment, while Co-expression Modules 2–9 (CM2-CM9) showed co-expression in the early (hourly) response phase post-sulfur treatment. Co-expression Modules 10–14 (CM10-CM14) were associated with the subsequent responses, serving as co-expressed gene groups involved in late (daily) responses post-sulfur treatment (Fig. 2).

### GO and KEGG pathway analysis to determine functions pointed out by co-expressed modules in response to sulfur treatment

**Fig. 3:**
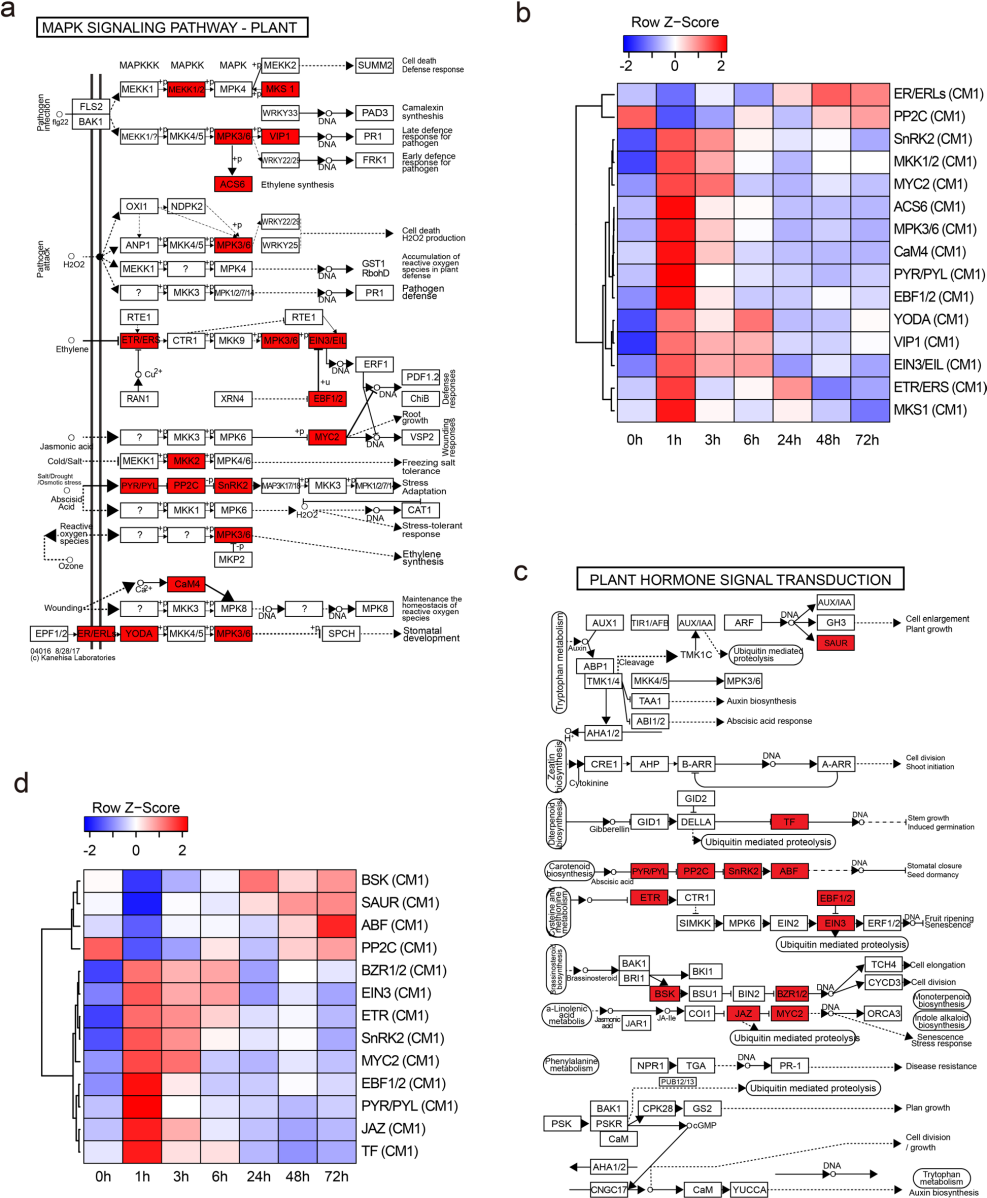
KEGG pathways enriched within CM1 showing early gene expression changes after sulfur treatment. (**a**) MAPK signaling pathway in plants, highlighting genes in CM1 with significant upregulation (red boxes) following sulfur treatment. (**b**) Heatmap of row Z-scores for CM1 genes in the MAPK signaling pathway across time points, where red and blue indicate upregulation and downregulation, respectively. (**c**) Plant hormone signal transduction pathway, with red box highlights indicating genes in CM1 exhibiting significant expression changes within an hour of sulfur treatment. (**d**) Heatmap of row Z-scores for CM1 genes in the plant hormone signal transduction pathway across time points, illustrating dynamic expression patterns. Parentheses indicate CM numbers. Parentheses indicate CM number, see Supplementary Table S5 for Solyc. IDs of genes used in the heatmaps in (b) and (d).

To determine the functions of each CM in sulfur-treated plants, we first conducted GO enrichment analysis, followed by KEGG pathway analysis to further elucidate the metabolic pathways involved^23^. GO enrichment analysis of CM1 revealed functional genes involved in protein autophosphorylation, intracellular signal transduction, and pattern specification processes (Supplementary Fig. S6a). To further explore the KEGG pathways in CM1, KEGG pathway gene set enrichment analysis was conducted, which identified significant pathways, including MAPK signaling, plant-pathogen interaction, and plant hormone signal transduction, indicating substantial changes in early co-expression in response to external signals and intracellular signaling (Fig. 2c). Specifically, MAPK signaling is a common mechanism in plant responses to sulfur treatment, with significant gene expression changes observed in pathogen interaction signaling, as well as responses to salt, drought, and osmotic stress. These genes displayed notable changes in the early signal recognition and transduction phase (Fig. 3a and b). Additionally, plant hormone signal transduction plays a crucial role in mediating responses to both internal and external environmental changes. CM1 enrichments were strongly associated with abscisic acid (ABA), jasmonate (JA), ethylene signaling, and auxin and brassinosteroid (BR) signaling, offering molecular insights into stress responses and plant growth following sulfur treatment (Fig. 3c and d). Interestingly, genes involved in ABA, JA, and ethylene signaling—typically related to stress responses—were transiently upregulated during the initial stress signaling induced by sulfur supply and subsequently returned to baseline. In contrast, genes associated with auxin, BR, and ABA signal transduction were induced from 24 h after treatment, indicating a temporal shift from stress response to growth and environmental adaptation (Fig. 3d).

**Fig. 4:**
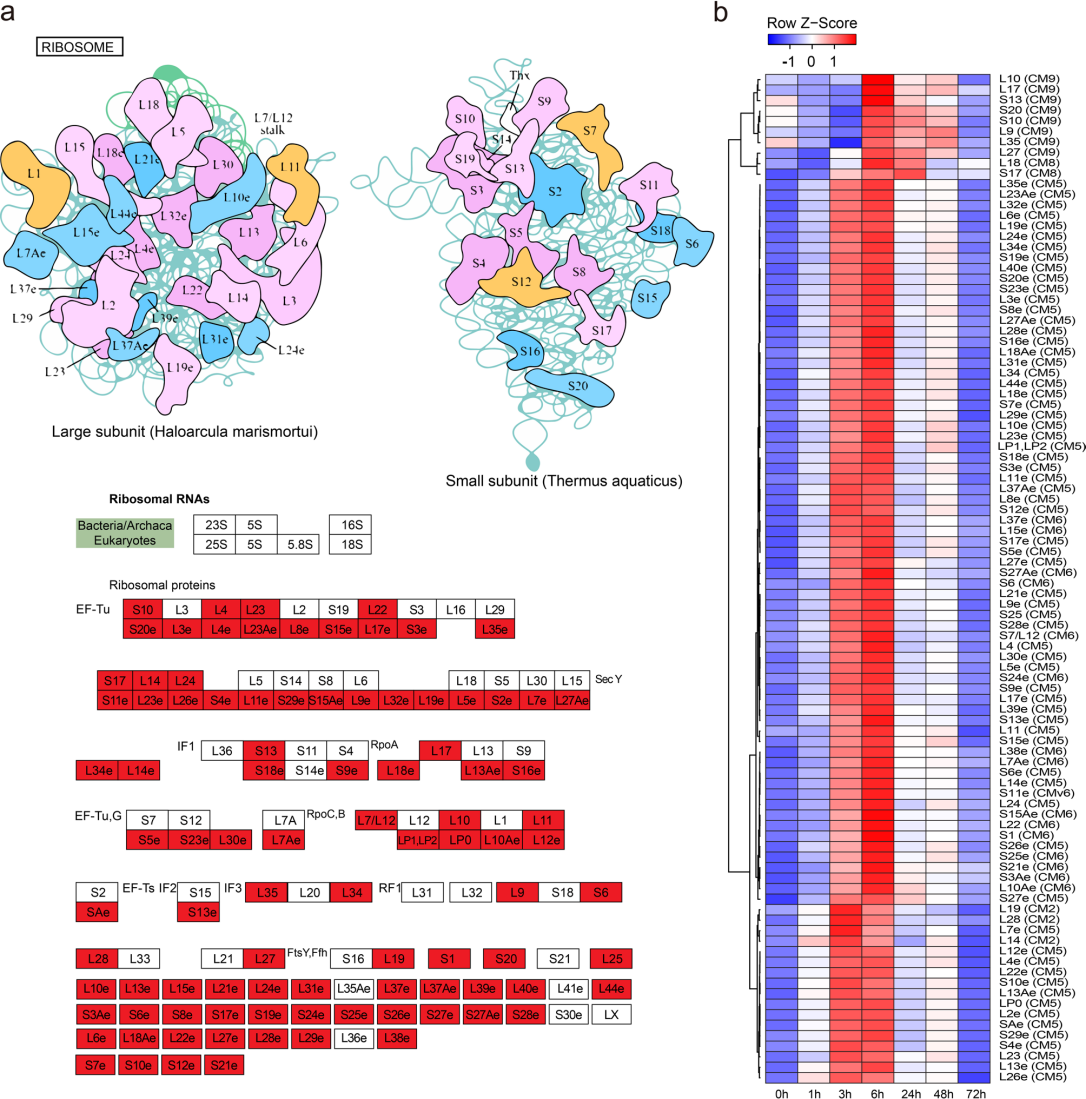
Ribosome KEGG pathways in CM5 and CM6 with dominant changes in gene expression 3–6 h after sulfur treatment. (**a**) Structural representation of the ribosome, showing the large subunit and the small subunit. Ribosomal RNAs and ribosomal proteins are annotated. Genes in CM5 and CM6 with significant expression changes are highlighted in red box, indicating their involvement in sulfur treatment responses. (**b**) Heatmap of row Z-scores for ribosomal protein genes in CM5 and CM6 across time points. Red and blue indicate upregulation and downregulation, respectively. Parentheses indicate CM number, see Supplementary Table S5 for Solyc. IDs of genes used in the heatmaps in (b).

**Fig. 5:**
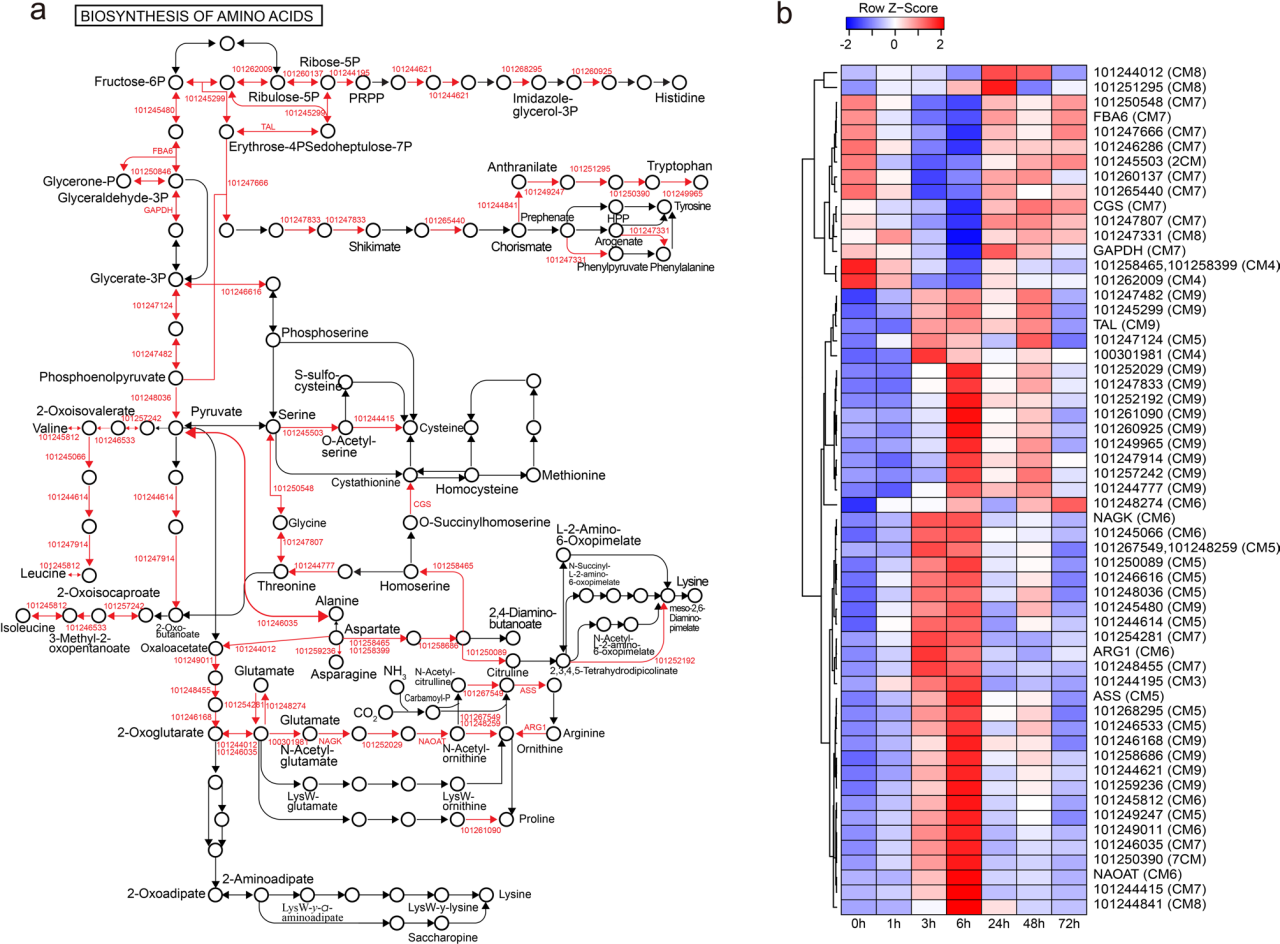
Biosynthesis of amino acid KEGG pathways in CM7 and CM9 showing dominant changes in gene expression 6 h after sulfur treatment. (**a**) Diagram of the biosynthesis of amino acid pathway, highlighting genes in CM7 and CM9 with significant expression changes. Genes with upregulation are indicated in red arrow on the pathway, showing their involvement in sulfur treatment responses. (**b**) Heatmap of row Z-scores for genes in CM7 and CM9 associated with amino acid biosynthesis across time points. Red color bar and blue color bar indicate upregulation and downregulation, respectively. Parentheses indicate CM number, see Supplementary Table S5 for Solyc. IDs of genes used in the heatmaps in (b).

CM2-CM9 showed a strong early hour-term response to sulfur treatment, with GO-enriched functions related to ribonucleoprotein complex biogenesis, nucleotide metabolism, and mRNA transport, which are critical for RNA synthesis, supporting protein translation and cellular activity. Additionally, GO-enriched functions were associated with mitochondrial and chloroplast organization, as well as carbohydrate derivative biosynthesis, which play vital roles in photosynthesis and stress adaptation (Supplementary Fig. S6b). KEGG pathway analysis revealed that co-expression enrichment of genes related to ribosome biogenesis and nucleocytoplasmic transport occurred across CM2, CM5, and CM6 (Fig. 2d). This co-expression pattern indicates expression changes initiated at 3 h after sulfur treatment and relayed to 6 h. In CM5 and CM6, approximately 70% of genes involved in ribosome formation were strongly upregulated between 3- and 6-hours following sulfur treatment (Fig. 4a and b; Supplementary Fig. S7a and b). Significant co-expression changes in aminoacyl-tRNA biosynthesis metabolic genes were observed in CM5 and CM7, while the co-expression of amino acid biosynthesis genes increased notably in CM5-CM9, particularly approaching 6 h after treatment (Supplementary Fig. S8c and d; Fig. 5a and b). These findings indicate that the biochemical pathways involved in amino acid and protein production were substantially enhanced in plant cells and tissues during the 3-to-6-hour post-sulfur treatment period.

Furthermore, significant co-expression changes in genes involved in carbon metabolism and photosynthesis pathways were observed in CM7, with relatively high expression alterations detected at 6 h after sulfur treatment. GO enrichment analysis further confirmed functional changes in processes such as carboxylic acid biosynthesis, rhythmic processes, and glucose metabolism (Supplementary Fig. S6b). Notably, GO term enrichment for rhythmic processes and responses to light stimulus suggested potential biases in hourly transcriptome analysis. To address this, the distribution of daily oscillating genes was examined across each CM, revealing that these genes accounted for less than 30% in all CMs except CM7, where 65% of genes were identified as oscillating (Supplementary Fig. S9, Supplementary Tables). This indicates that the significant co-expression of genes in CM7 contains a common feature of hourly transcriptome changes.

**Fig. 6:**
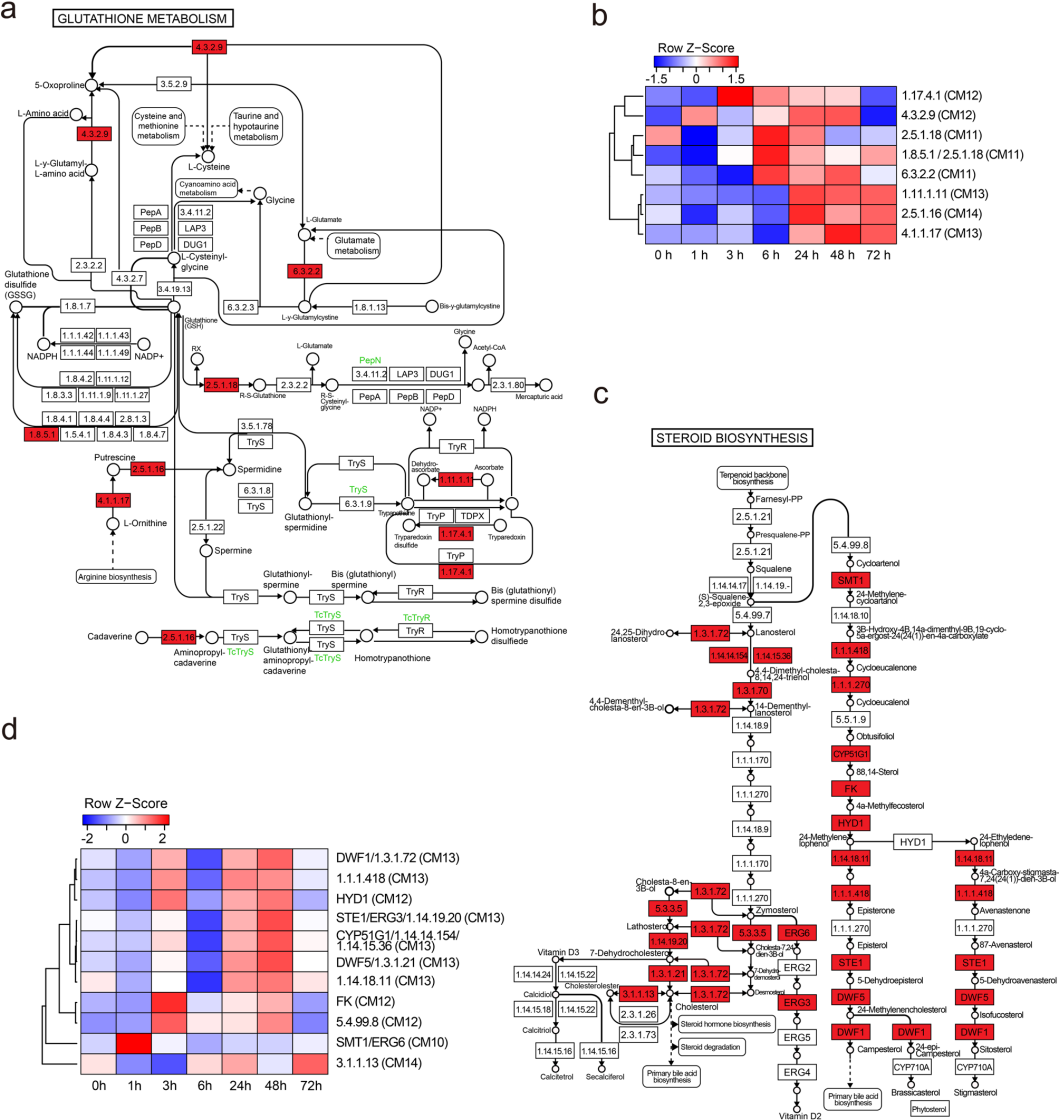
Glutathione metabolism and steroid biosynthesis KEGG pathways showing co-expression changes in daily response after sulfur treatment. (**a**) Diagram of the glutathione metabolism pathway, with upregulated genes in CM11, CM12, CM13, and CM14 highlighted in red color boxes. (**b**) Heatmap of row Z-scores for glutathione metabolism genes across time points. Red color bars represent upregulation, but blue color bars indicate downregulation, respectively, with genes annotated by enzyme classifications and CM numbers. (**c**) Steroid biosynthesis pathway with significant gene expression changes shown in red, indicating co-expression responses to sulfur treatment. (**d**) Heatmap of row Z-scores for steroid biosynthesis pathway genes, highlighting dynamic expression patterns across time points. Parentheses indicate CM number, see Supplementary Table 5 for Solyc. IDs of genes used in the heatmaps in (b).

GO enrichment analysis of CM10-CM14 revealed involvement in plant hormone response, cell division promotion, and steroid biosynthesis, suggesting their roles in the later stages (daily-term) of the plant’s response to sulfur treatment (Supplementary Fig. S6c). To further investigate the KEGG pathways in CM10-CM14, gene set enrichment analysis revealed significant roles in protein folding, cellular signaling, and stress response, highlighting pathways such as ‘Protein processing in endoplasmic reticulum,’ ‘Glutathione metabolism,’ and ‘Plant hormone signal transduction.’ (Fig. 2e). The CM11-CM13 showed significant changes in gene expression related to glutathione metabolism, indicating that glutathione plays a crucial role not only in the detoxification of reactive oxygen species (ROS) during stress responses but also in the regulation of redox signaling, gene expression, and cellular defense mechanisms (Fig. 6a and b). The CM12 showed significant changes in gene expression related to BR biosynthesis and other steroid-related metabolic processes. Steroids are vital for plant immune responses and growth regulation (Fig. 6c and d). Additionally, cell division processes, such as motor protein activity and homologous recombination, were significantly observed in CM12 and CM14 (Supplementary Fig. S10a and b). These changes in gene expression indicate enhanced cell division following the initial sulfur treatment response.

### Sulfur treatment enhances immunity, improves drought tolerance, and increases yields in tomato

**Fig. 7:**
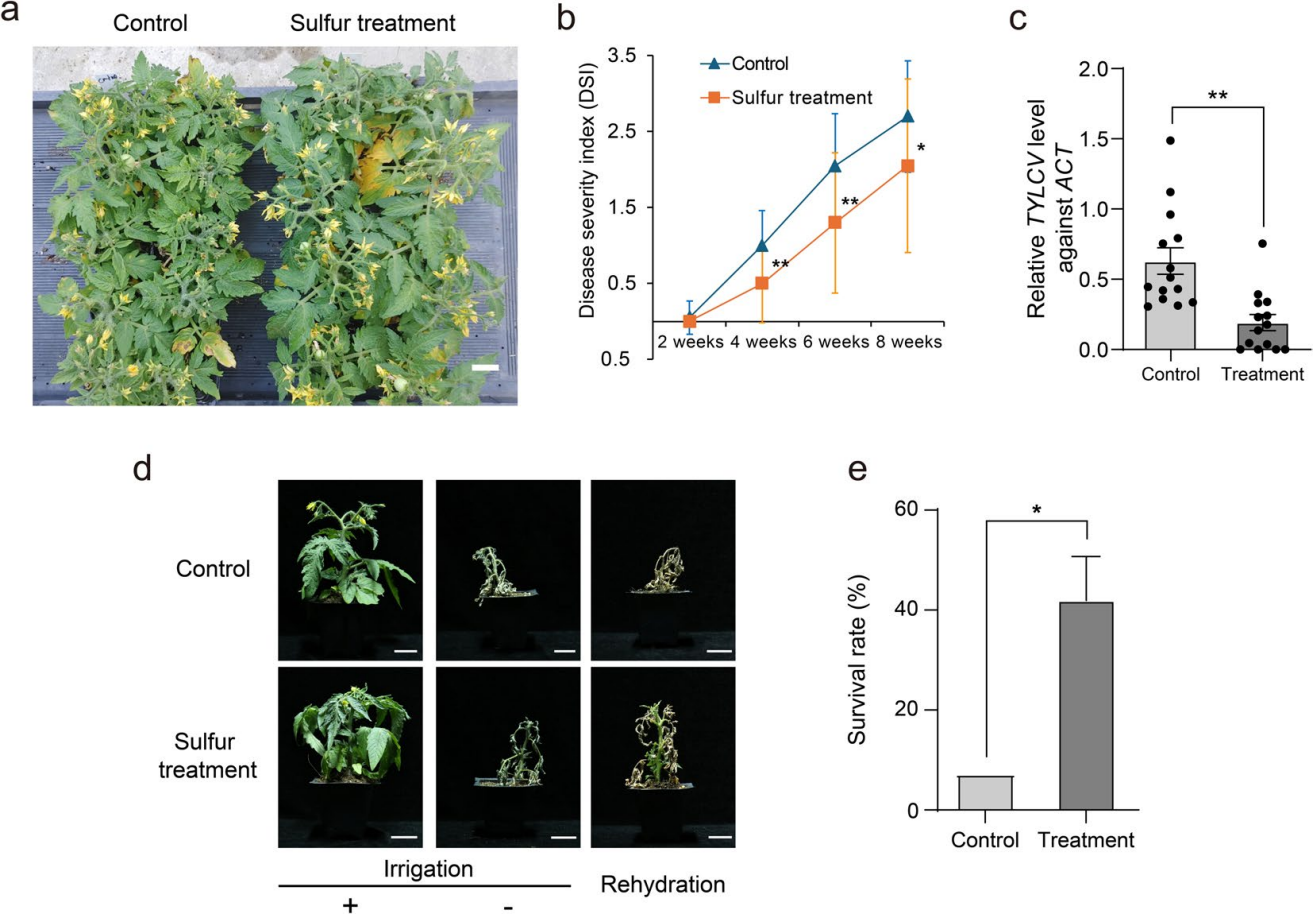
Quantitative comparison of TYLCV symptoms and resilience to drought stress in plants from sulfur treatments. (**a**) Representative plants showing TYLCV symptoms at 4 weeks after Agrobacterium infiltration with TYLCV infectious DNA clone in control (left) and sulfur-treated (right) plants. (**b**) Line graph comparing Disease Severity Index (DSI) between control and sulfur-treated groups, assessed at 2-week intervals using 20 independent plants per group; DSI criteria described in Supplementary Fig. 11. (**c**) Relative TYLCV accumulation in control and sulfur-treated plants at 6 weeks post-inoculation, measured by qPCR using TYLCV-specific primers and normalized to β-actin Ct values. (**d**) Representative images showing plant responses to irrigation and rehydration following drought stress in control (untreated) and sulfur-treated plants. Scale bar: 5 cm. (**e**) Bar graph illustrating the survival rate (%) of control and sulfur-treated plants after rehydration following drought stress. Statistical significance (*p < 0.05, **p < 0.01) was determined using Student’s *t*-tests, with the 0 mg/L sulfur treatment as the control. Scale bar, 2 cm.

Based on transcriptomic analysis revealing significant changes in gene expression following sulfur treatment, we designed experiments to investigate its effects on tomato plant immunity, stress tolerance, and fruit productivity. To evaluate the role of sulfur in enhancing immunity, we validated its effect on TYLCV resistance through an infection assay using Agrobacterium-mediated delivery of TYLCV DNA. Sulfur-treated plants consistently exhibited lower disease severity and viral titers, confirming a sustained protective effect during the early to mid-phase of infection (Fig. 7a–c). The disease severity index (DSI) was monitored biweekly, and viral accumulation was quantified at 6 weeks post-inoculation (Fig. 7b, c, and Supplementary Fig. S11).

To investigate the role of sulfur in improving stress tolerance, we treated tomato plants with 0.4 mg/L sulfur and subjected the plants to controlled drought stress. After rehydration, we measured the plants’ recovery rates and found that sulfur-treated plants had significantly higher tolerance to drought than the untreated controls (Fig. 7d and e). Notably, 3,3’-diaminobenzidine (DAB) staining at 8 days after dehydration revealed reduced H₂O₂ accumulation in sulfur-treated plants, supporting their enhanced oxidative stress tolerance under drought conditions (Supplementary Fig. S12). This observation is consistent with transcriptomic data showing upregulation of genes involved in glutathione metabolism and redox regulation, which play key roles in ROS detoxification.

**Fig. 8:**
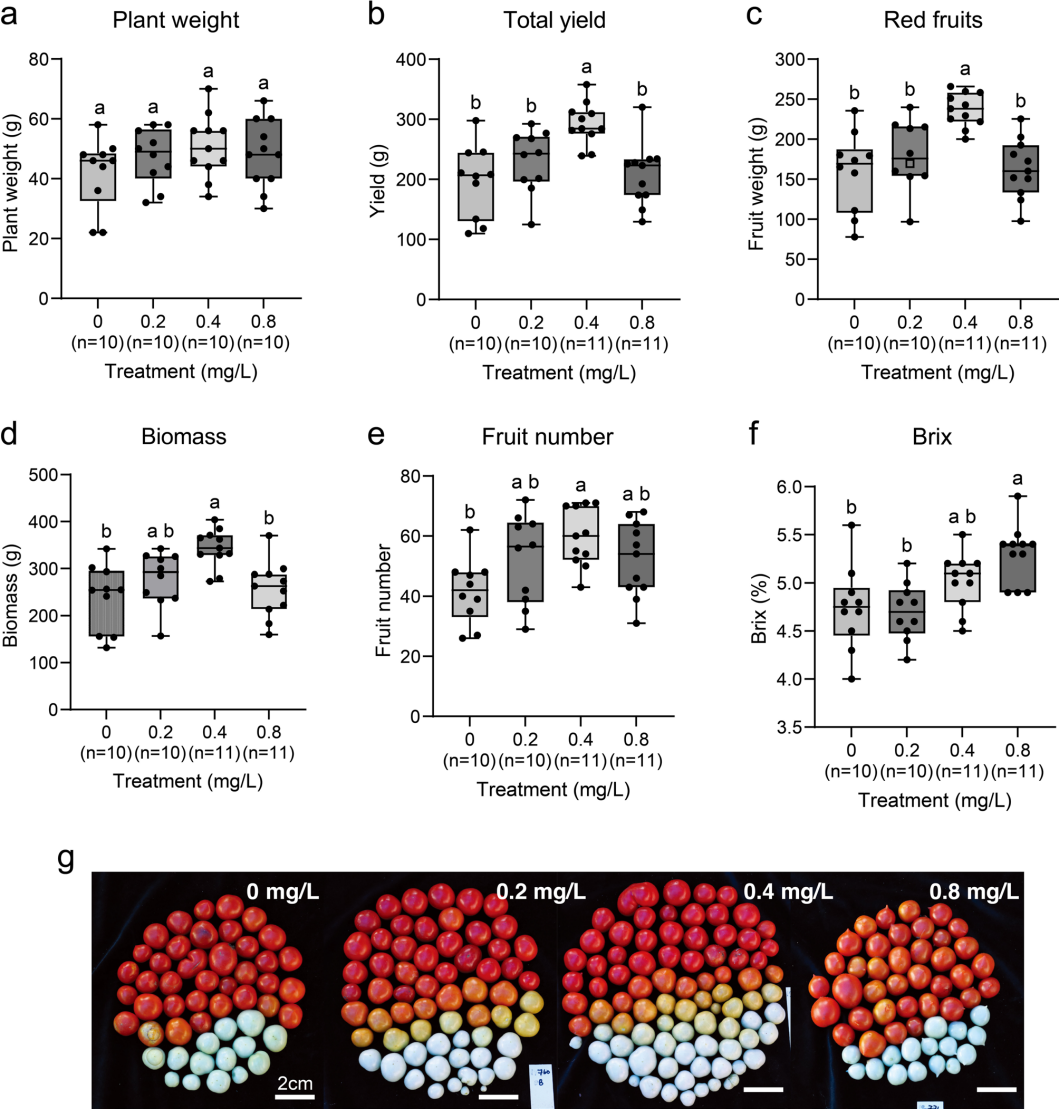
Quantitative comparison of tomato yield-related traits in response to different sulfur concentrations. (**a**–**f**) Quantification and comparison of plant weight (a), total yield (b), red fruit weight (c), biomass (d), fruit number (e), and Brix (f) under 0, 0.2, 0.4, and 0.8 mg/L sulfur treatments. Biomass represents the combined weight of the plant and total fruits. Statistical significance, assessed by one-way ANOVA followed by Tukey’s HSD test, is indicated by different letters (*p* < 0.05). Box plots show the 25th and 75th percentiles (box boundaries), the median (bold line), and whiskers extending to 1.5× the interquartile range (IQR). Each dot represents the value from individual plant. n, the number of replicates. (**g**) Representative images comparing harvested fruits sorted by sulfur treatment. Scale bar: 2 cm.

To evaluate the effects of sulfur fertilization on plant growth, tomato yield, and fruit quality, we conducted the performance trials. In plant growth, plant weight increased proportionally with sulfur application, although statistical analysis revealed no significant differences between treatment groups (Fig. 8a). The 0.4 mg/L sulfur treatment resulted in the most pronounced improvement in mature and total fruits yield, showing a statistically significant increase compared to the water-treated 0 mg/L control (Fig. 8b, c, g). Fruit number remained consistent across all treatments up to 0.8 mg/L sulfur concentrations (Fig. 8e).

In the second yield trial, the average red fruit and total fruit yield were highest in the 0.4 mg/L sulfur treatment, though not statistically significant. Plant weight, representing vegetative growth, showed a significant increase under sulfur treatment (Supplementary Fig. S13). Biomass was evaluated by combining fruit yield and plant weight, and both trials confirmed that the 0.4 mg/L treatment supported the most balanced growth and productivity (Fig. 8d and Supplementary Fig. S13c).

Fruit quality, measured through sugar content (Brix), improved significantly in the 0.4 mg/L and 0.8 mg/L treatments, with the 0.8 mg/L treatment achieving the highest sugar levels (Fig. 8f, Supplementary Fig. S13f). Both trials confirmed that the optimal sulfur dosage of 0.4 mg/L to 0.8 mg/L sulfur concentration maximizes yield and quality, while excessive application, such as 1.6 mg/L sulfur, diminishes these benefits.

## Discussion

This study integrates transcriptomic and phenotypic analyses to elucidate how sulfur treatment enhances tomato plant growth, stress tolerance, and productivity. By linking gene expression changes to phenotypic traits such as improved immunity, drought resilience, and fruit harvest, the research establishes sulfur’s dual role as a nutrient and signaling modulator with the potential to optimize agricultural outcomes. Most of all, while sulfur treatment at 0.4 mg/L enhanced plant growth and metabolic activity, higher concentrations resulted in growth inhibition, indicating potential metabolic trade-offs and suggesting a dose-dependent effect.

Most of all, appropriate sulfur application (as shown in this study using elemental sulfur at 0.4 mg/L) enhanced plant growth and metabolic activity, whereas higher concentrations led to growth inhibition, indicating potential metabolic trade-offs and a dose-dependent effect.

Sulfur treatment induces transcriptional changes that prepare plants for environmental challenges by regulating stress and growth pathways. Early responses include a transient decrease in sulfur transporter gene expression, likely as feedback to prevent excess sulfur uptake. This is followed by activation of MAPK signaling and hormone-regulated processes, such as JA and ABA signaling, within the first hour after sulfur treatment. These early adjustments enable plants to handle biotic and abiotic stresses, as reflected in reduced TYLCV incidence during the resistance period over eight weeks, linked to upregulated genes involved in plant-pathogen interactions and secondary metabolism, reinforcing sulfur’s role in plant defenses^24,25^.

Additionally, sulfur treatment, as a supplier of a vital nutrient, enhances translation capacity and amino acid biosynthesis, as evidenced by the upregulation of ribosome biogenesis and aminoacyl-tRNA synthetase pathway genes. This enhanced translational activity supports rapid cellular responses, as observed in previous studies where sulfur treatment led to increased biomass and improved metabolic activity^26^. Phenotypically, this correlates with accelerated stem, root, and leaf growth, supported by transcriptomic evidence of enhanced protein synthesis associated with increased cell division and expansion. This growth response follows the later upregulation of growth hormone signaling genes—such as those involved in auxin and BR signaling pathways—observed 24 h after sulfur treatment, which likely promotes sustained developmental processes at the molecular level^13,27^. Simultaneously, amino acid biosynthesis pathways provide resources for proteins critical in stress adaptation, bolstering resilience. While sulfur supports growth and stress tolerance, the transient nature of its immune effects suggests periodic sulfur application may sustain long-term benefits^28,29^.

Furthermore, long-term transcriptomic changes revealed shifts in genes linked to stress tolerance and growth. Upregulated genes involved in glutathione metabolism and redox regulation are molecularly associated with improved drought resilience, as sulfur-treated plants showed higher recovery rates after rehydration^30^. The upregulation of growth-related hormone signaling genes—including auxin, BR, and ABA—from 24 h post-treatment supports enhanced plant growth and stress tolerance during the late phase. These findings underscore sulfur’s role in plant growth and abiotic stress adaptation by reducing oxidative stress and maintaining cellular response.

Sulfur treatment enhances growth and yield through molecular mechanisms, with gene expression changes suggesting potential pathways. Late transcriptomic responses included upregulated genes linked to glutathione metabolism, steroid biosynthesis, and cell division and growth, contributing to sustained growth and resilience for long-term adaptation^31,32^.

These findings demonstrate sulfur’s ability to optimize plant responses over time, improving productivity and resilience against diverse environmental conditions. By bridging molecular mechanisms with practical outcomes, the study shows how sulfur-induced transcriptional changes enhances growth, immunity, and stress tolerance. Future research should refine application protocols, explore long-term stability of molecular effects, and evaluate sulfur’s role across diverse crop systems. Understanding sulfur’s interplay with other nutrients may unlock synergistic effects, broadening its role in sustainable agriculture. By integrating molecular and phenotypic data, this study lays a foundation for strategies to enhance crop resilience and productivity in response to agricultural challenges, positioning sulfur as both an essential nutrient and a regulator of plant adaptation.

## Materials and methods

### Plant materials and growth conditions

The plants were grown under standard greenhouse conditions (16-hour light/8-hour dark photoperiod, temperature 20–25 °C). Seeds of the cultivar Micro-Tom (accession no., TOMJPF00001) obtained from the TOMATOMA database were used for all experiments. The seeds were first germinated and grown for 4 weeks in a 50-cell plastic tray and then transplanted into pots (a top diameter of 15 cm, a bottom diameter of 10.3 cm, and a height of 13.5 cm). All the plants were grown under drip irrigation and standard fertilizer regimes.

### Elemental sulfur treatment, growth assessment, and sulfur content assay

Nine Days after germination (DAG) plants were transplanted into pots with a top diameter of 15 cm, a bottom diameter of 10.3 cm, and a height of 13.5 cm. The ‘Modoo Ssak’ pure elemental sulfur solution (Nara Bio, Korea) was prepared at concentrations of 0 mg/L, 0.2 mg/L, 0.4 mg/L, 0.8 mg/L, and 1.6 mg/L. Each week for five weeks, 50 mL of the solution was either sprayed onto the leaves and plants or drenched into the soil to allow absorption through the roots. Plant growth measurements, including stem length, root length, and plant weight, were conducted weekly to evaluate the effectiveness of the treatments.

To measure the sulfur content in plant tissues, plants at 34 DAG were subjected to two experimental approaches. For short-term response analysis, 25 mL of a 0.4 mg/L sulfur solution was applied to pots, and aerial tissues were sampled at 0 h (control; h), 1 h, 3 h, 6 h, 12 h, 24 h, 48 h, and 72 h after treatment. For long-term analysis, aerial tissues were collected at 34 DAG following four sulfur treatments applied every six days, with 25 mL of solutions at 0, 0.2, 0.4, and 0.8 mg/L concentrations. The aerial plant tissues were dried at 50 °C for 8 h, ground, and digested in nitric acid using a Microwave Digestion System (Ethos EASY Advanced, Milestone). Sulfur content was quantified using Inductively Coupled Plasma Optical Emission Spectrometry (OPTIMA 8300DV, PerkinElmer). A minimum of 12 replicates were used for each analysis to ensure statistical reliability.

### RNA sequencing

To perform time-dependent mRNA-seq profiling after pure elemental sulfur treatment (Modoo Ssak, Nara Bio), plants grown for 21 days in 50-cell plastic trays were treated by spraying 250 ml of a 0.4 mg/L sulfur solution onto one 50-cell tray. Plant tissues, excluding roots, were sampled at 0 h (control), 1 h, 3 h, and 6 h after the treatment for short-term (hourly) profiling, and at 24, 48, and 72 h for long-term (daily) profiling. The aerial parts of the plants were collected and immediately frozen in liquid nitrogen. Three biological replicates were performed for each time point.

Total RNA was extracted using the RNeasy^®^ Plant Mini Kit (QIAGEN, Valencia, CA, USA), and DNA was removed using DNase I (QIAGEN). Paired-end 150 bp RNA sequencing was performed using the TruSeq Stranded mRNA Library Prep Kit and the Illumina NovaSeq 6000 platform, generating more than 5 GB of data per sample.

The quality of raw mRNA-seq reads was assessed using FastQC v0.11.9 (https://www.bioinformatics.babraham.ac.uk/projects/fastqc/) and MultiQC v1.11^33^. Low-quality reads were filtered using Trimmomatic v0.39^34^. Trimmed reads were mapped to the *S. lycopersicum* reference genome (SL4.0) and gene annotation (ITAG4.1) using STAR v2.7.9a^35^and transcripts per million (TPM) values were calculated using RSEM (v1.3.1)^36^.

Expression profiles were analyzed using DESeq2 (v1.26.0)^37^ to identify differentially expressed genes (DEGs) after the elemental sulfur (Modoo Ssak, Nara Bio) treatment. The criteria for identifying DEGs were set as mean TPM ≥ 1, log2 fold change ≥ ± 1, and FDR < 0.1. The analysis was performed for short-term responses (0 h, 3 h, 6 h, 24 h) and daily responses (0 h, 24 h, 48 h, 72 h), respectively. This resulted in the identification of 2,754 DEGs in the short-term response and 268 DEGs in the daily response. To further analyze the gene expression patterns over time, we performed k-means clustering using R’s Mfuzz package^38^. The clustering resulted in the categorization of genes into 10 clusters for short-term responses and 8 clusters for daily responses.

Genes with an average TPM value ≥ 3 were filtered based on the MAD (Median Absolute Deviation) of their log2(TPM + 1) values, selecting the top 75% with the highest variability (a total of 12,062 genes). Weighted gene co-expression network analysis (WGCNA) was performed on the selected genes using the WGCNA R package^39^. Sample clustering confirmed the absence of outliers, and the optimal soft threshold parameter was determined to be 15 based on scale independence and mean connectivity analysis. The minimum module size (min Module Size) was set to 100, and the merging threshold (merge Cut Height) was set to 0.05, resulting in 14 distinct modules. Based on eigengene expression patterns, CM1 was classified as signal transduction, CM2-9 as early responses, and CM10-14 as post-early responses, which were grouped into meta-modules.

To identify diurnal oscillation genes within the meta-modules, we referred to Zhang et al. (2023)^40^. Genes with at least a two-fold difference in TPM values at specific time points were redefined as oscillation genes. These genes were then compared with the meta-modules to analyze their number and distribution in each module.

### Functional enrichment analysis of cluster genes

To understand the biological function of the meta-modules, we performed GO term enrichment analysis using both the topGO (v2.50.0) and the clusterProfiler (v 4.6.2) R packages ^41,42^. Biological process (BP) GO terms with p-value < 0.05 in all modules or in each module were selected and visualized using the ggplot2 R package.

To identify metabolic pathways containing meta-modules, KEGG pathway enrichment analysis was performed using the enrich KEGG function of the cluster Profiler R package^23^. KEGG pathways with p-value < 0.05 in all modules or in each module were selected, and the related genes in the enriched KEGG pathways were visually represented using the pathview R package^43^. In addition, the expression patterns of genes included in the meta-modules were visualized using the heatmap2 R package. KEGG pathway data used in this study were provided with permission from Kanehisa Laboratories.

### Drought resistance assay and ROS staining

Four-week-old plants were grown in pots (2.8 cm top diameter, 1.9 cm bottom diameter, 5.5 cm height) under 16/8 h light/dark conditions in a greenhouse. Elemental sulfur (25 mL of 0.4 mg/L) was applied to the soil on days 3 and 10 after transplanting. Control plants received the same volume of distilled water. After the second sulfur treatment, drought stress was induced by completely withholding water for 14 days, followed by 14 days of rehydration. Uniform and healthy plants with similar height and width were selected for the experiment. To minimize positional effects during natural dehydration, plant positions were randomly assigned within the greenhouse. Plants that produced new leaves 14 days after rehydration were defined as surviving plants. The experiment was repeated more than three times with at least 28 plants per treatment group. To evaluate ROS accumulation, plants were subjected to the same drought assay. Leaves were stained at 2 and 8 days after the second sulfur treatment using DAB for H₂O₂ as previous study^44^. Stained leaves were decolorized in boiling 96% ethanol and stored in fresh ethanol at room temperature until imaging.

### TYLCV disease induction and quantification

TYLCV inoculation and quantification were conducted based on the method of a previous study^45^with partial modifications. *Agrobacterium tumefaciens* strain GV3101 carrying the infectious TYLCV clone (pCAMBIA3301-TYLCV) was grown in LB medium supplemented with kanamycin (50 µg/mL), rifampicin (100 µg/mL), and gentamycin (25 µg/mL) at 28 °C with shaking. Agrobacterium cells were collected by centrifugation, resuspended in infiltration buffer (10 mM MgCl₂, 200 µM acetosyringone, 10 mM MES) to an OD₆₀₀ of 0.7–0.8, and incubated at room temperature for 2 h. The agrobacterial suspension was then infiltrated into the abaxial side of fully expanded leaves using a needleless syringe. TYLCV symptom progression was monitored every two weeks up to 8 weeks post-inoculation. Disease severity was evaluated using a visual Disease Severity Index (DSI): 0 = no symptoms, 1 = mild leaf curling, 2 = moderate curling and plant stunting, and 3 = severe stunting and yellowing. At 6 weeks post-inoculation, three young leaves from each plant were sampled for TYLCV quantification. Genomic DNA was extracted using DNeasy mini kit (QIAGEN, Germany), and 100 ng of DNA was used for quantitative PCR with TYLCV-specific primers targeting the TYLCV C1 gene (185 bp) and β-actin as the internal control. The reaction was performed using SYBR Green I on a Chromo4 real-time PCR system (Bio-Rad, USA), and relative viral accumulation was calculated based on Ct values.

### Yield trials

To evaluate the effects of the elemental sulfur treatment concentrations on fruit yield, 28 DAG plants were transplanted into pots with a top diameter of 15 cm, a bottom diameter of 10.3 cm, and a height of 13.5 cm. One week after transplanting, the sulfur was applied at different concentrations weekly over a total period of 6 weeks. For the first yield assessment, the elemental sulfur was applied at concentrations of 0, 0.2, 0.4, and 0.8 mg/L, while for the second yield assessment, it was applied at concentrations of 0, 0.4, 0.8, and 1.6 mg/L, with 50 mL per pot for each treatment. Yield measurements were taken when 75% of the whole fruit was ripe, and we measured plant weight, total yield, number of green fruits, number of red fruits, total number of fruits, and Brix.

## Supplementary Information

Below is the link to the electronic supplementary material.

Supplementary Material 1

Supplementary Material 2

## Data Availability

All datasets supporting the conclusions of this article are included in the article and supplementary files. The RNA-seq raw reads were deposited in the NCBI Sequence Read Archive (SRA) under BioProject accession PRJNA1209904.
