# Supplementary material for: Molecular and phenotypic insights into sulfur’s role in enhancing tomato plant growth, stress tolerance, and productivity
Source: Sci Rep. 2025 Sep 25;15:32773. doi: 10.1038/s41598-025-17645-3 (PMC12464245; doi:10.1038/s41598-025-17645-3)
Supplement: Supplementary file 2 — Supplementary Material 2 [file 41598_2025_17645_MOESM2_ESM.docx]

Supplementary Information for

**Molecular and Phenotypic Insights into Sulfur's Role in Enhancing Tomato Plant Growth, Stress Tolerance, and Productivity**

Junwoo Lee^1,†^, Jung Heo^1,†^, Eun Song Lee^1^, Hye-yeong Kang^1^, Smita Mirsyad Warsadiharja^1^, Keunhwa Kim^1^, Yousun Chae^1^, Aditya Nurmalita Pervitasari^2^, Ryza A. Priatama^3^, Young Koung Lee^3^, Il Ho Kim^4, *^, and Soon Ju Park^1,*^

^†^J.W.L and H.J. contributed equally to this work.

* Correspondence should be addressed to I.H.K. ([79421515@naver.com](mailto:79421515@naver.com)) and S.J.P. ([sjpark75@gnu.ac.kr](mailto:sjpark75@gnu.ac.kr)).

**This PDF includes**

Supplementary Figure S1-S13 with legends


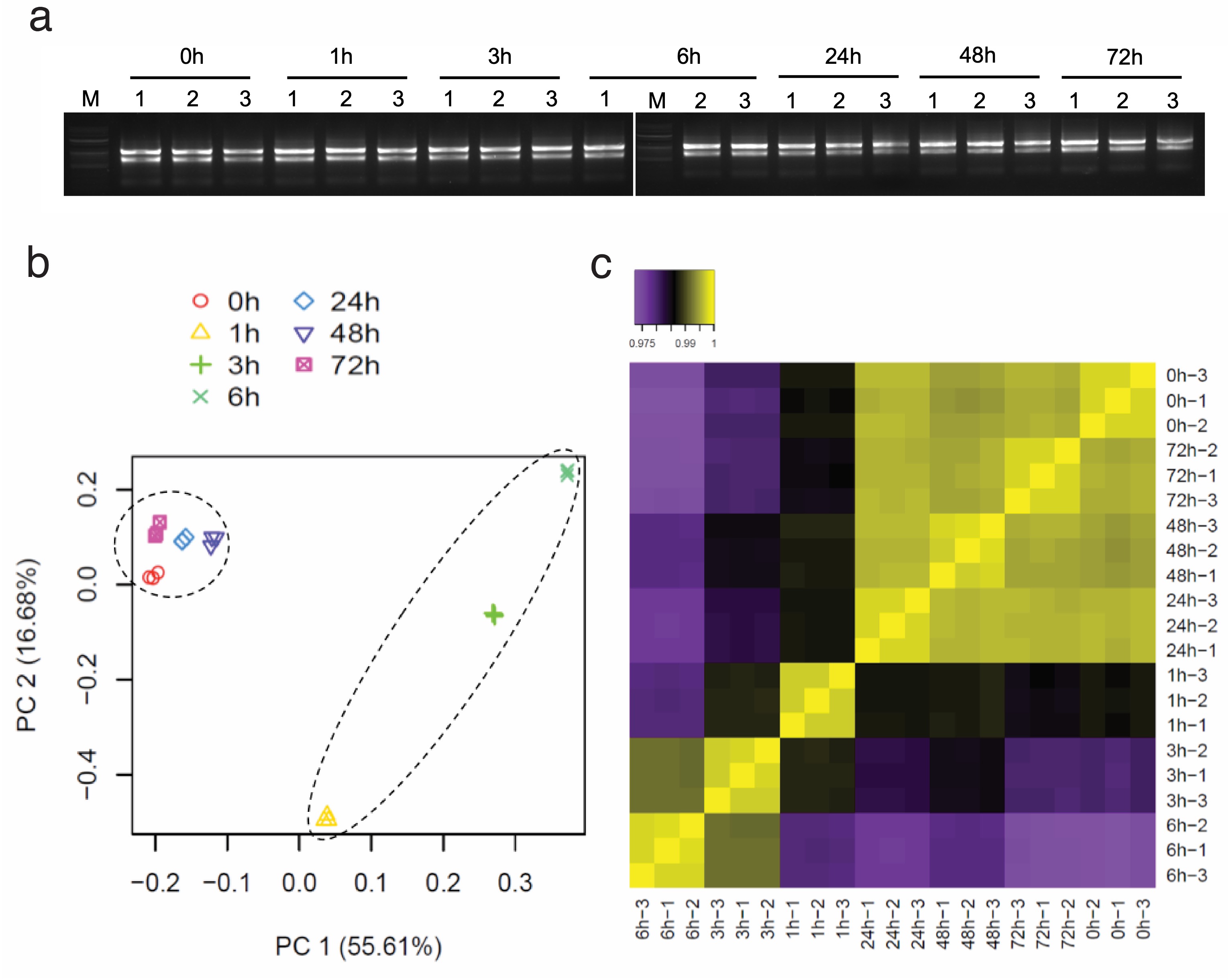


**Supplementary Figure S1. Expressional correlation between RNA seq profiling at different time points following elemental sulfur treatment.**

**(a)** Gel electrophoresis results showing the RNA quality and integrity across six time points (0, 1, 3, 6, 24, 48, and 72 hours) after elemental sulfur treatment. Each time point includes three replicates (labeled 1, 2, and 3), with the ladder (M) shown on the left. h = hour.

**(b)** Principal component analysis (PCA) plot depicting the clustering of RNA sequencing data at six time points following elemental sulfur treatment. Symbols of the same shape and color represent replicates, and dashed circles group profiles that are like each other.

**(c)** Heatmap of correlations among RNA sequencing profiles from different time points and replicates. Strong correlations within the same time point groups confirm reproducibility, while varying correlations indicate dynamic transcriptional changes during the early (hourly) responses to sulfur treatment, with relatively closer correlations observed in the late (daily) responses to elemental sulfur treatment.

**
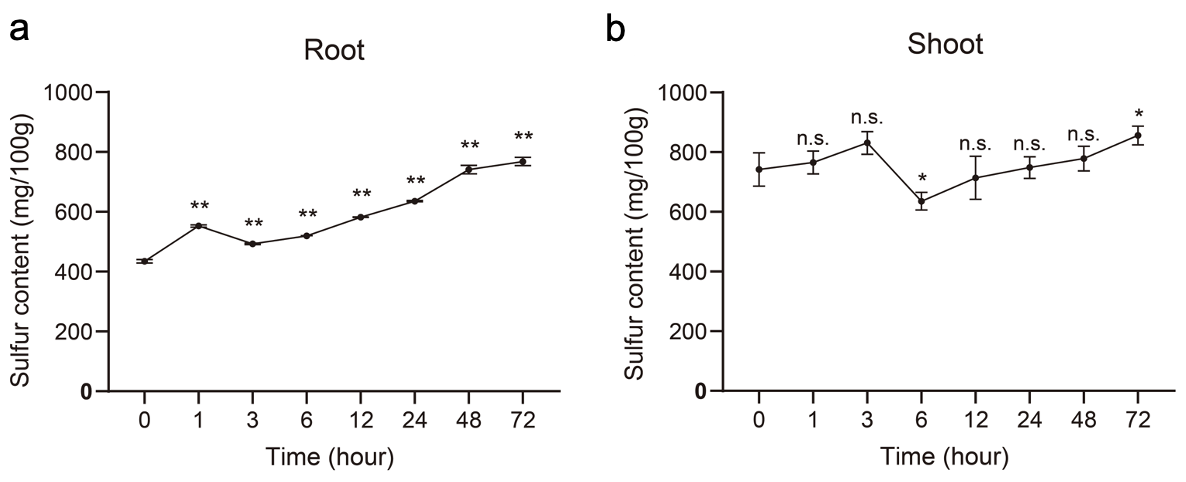
**

**Supplementary Figure S2. Quantification of the amount of endogenous sulfur in plants over time after elemental sulfur treatment.**

**(a-b)** Changes in dissolved sulfur content (mg/100g) in root tissues (a) and in aerial tissues (b) of tomato plants from 0 to 72 hours after elemental sulfur was applied to the soil at a concentration of 0.4 mg/L. Three-week-old plants had been used for these measurements for three days after sulfur treatment. Asterisks indicate significant differences compared to the content of 0 hour as control: *p < 0.05, ** p < 0.01, n.s. = not significant. Error bars represent standard deviation (n = 3 per treatment group). Statistical significance was determined using Student’s *t*-tests.

Elemental sulfur was applied to the soil in the pots to ensure that it did not come into direct contact with the aerial parts of the plants. This method allowed the sulfur to be absorbed by the roots and delivered to the rest of the plant.

**
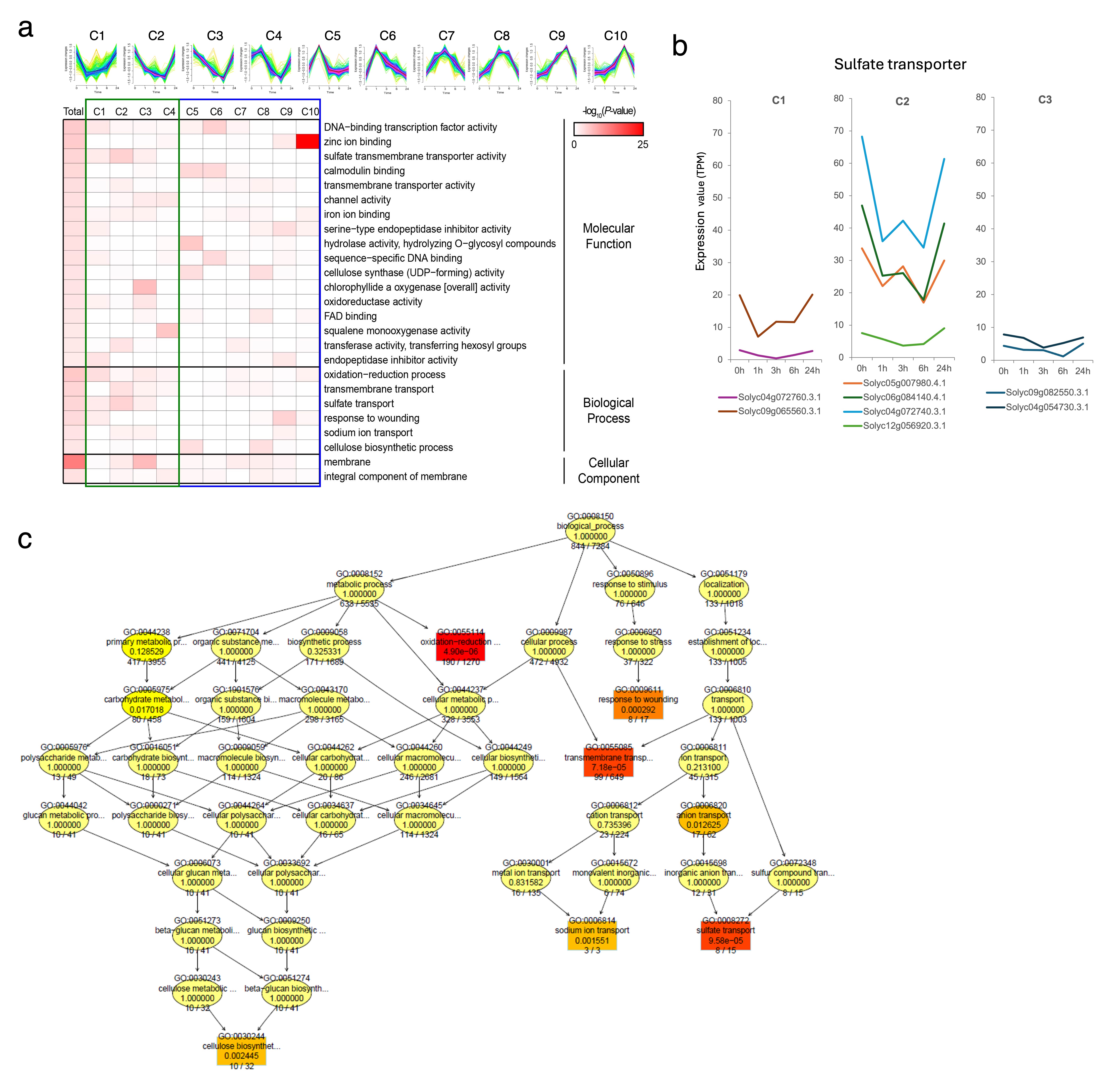
**

**Supplementary Figure S3. GO enrichment analysis of DEG genes in early (hourly) response following sulfur treatment.**

**(a)** Heatmap showing the GO enrichment analysis of DEGs categorized into three main GO domains: Molecular Function, Biological Process, and Cellular Component. The enrichment levels (-log_10_P value) are visualized with color intensity (red indicating higher enrichment and white indicating no enrichment). Green line box indicates the "reduced early response" clusters (C1–C4) and blue line box indicate the "increased early response" clusters (C5~10).

RNA sequencing data was collected at hourly intervals (0, 1, 3, 6, and 24 hours) after sulfur treatment, identifying 2,754 differentially expressed genes (DEGs; Supplementary Table S2). These DEGs were grouped into 10 clusters (C1–C10) using K-means clustering based on their expression patterns. Clusters were ordered by the time of peak gene expression, starting with the time of the sulfur treatment (top).

**(b)** Line plots displaying the temporal expression profiles of sulfate transporter genes across clusters C1, C2, and C3. An individual-colored line represents the hourly gene expression dynamics of the sulfate transporter gene.

**(c)** The GOrilla analysis visualizes enriched GO terms in ranked gene lists, emphasizing the hierarchical structure and relationships between the enriched GO terms associated with DEGs. Nodes (colored boxes) represent individual GO terms, with yellow-to-red color reflecting the degree of enrichment, where red indicates the highest significance. Arrows illustrate the hierarchical connections between GO terms, showing functional relationships or shared genes within the GO terms.


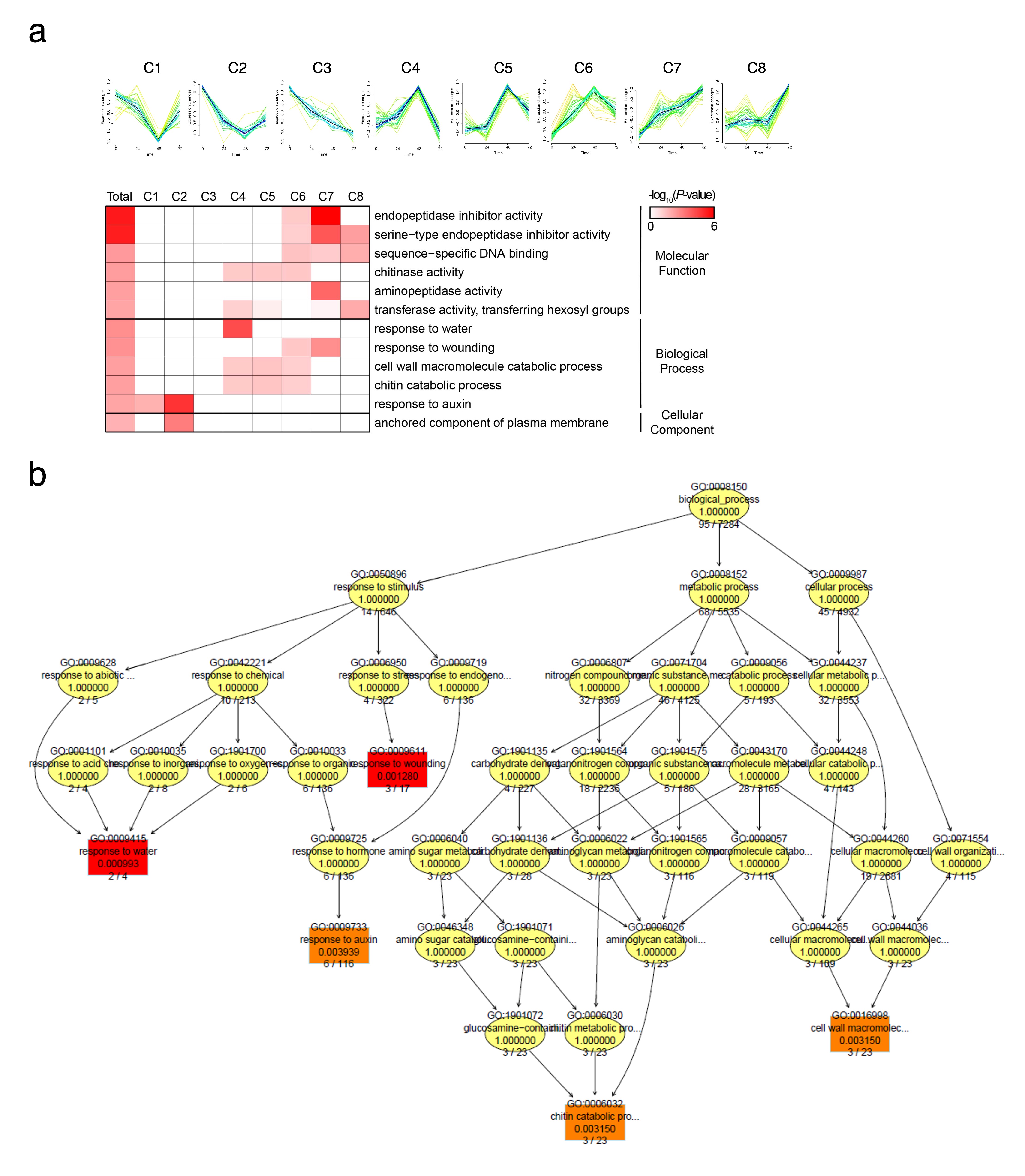


**Supplementary Figure S4. GO enrichment analysis of DEG genes in late (daily) response after sulfur treatment.**

**(a)** Heatmap displaying the GO enrichment analysis of DEGs categorized into three primary GO domains: Molecular Function, Biological Process, and Cellular Component. The enrichment levels (-log_10_P value) are represented by color intensity, with red indicating higher enrichment and white representing no enrichment. The clusters (C1–C8) were generated based on RNA sequencing data collected over a long-term response to sulfur treatment. Each cluster reflects genes with distinct expression patterns over time.

RNA sequencing data was collected at daily intervals (0, 1, 2, and 3 days) after sulfur treatment, identifying 268 DEGs (Supplementary Table S3). These DEGs were grouped into 8 clusters (C1–C8) using K-means clustering based on their expression patterns. Clusters were ordered by the time of peak gene expression, starting with the time of the sulfur treatment (top).

**(b)** GOrilla analysis visualizing enriched GO terms in ranked gene lists, highlighting hierarchical relationships between enriched GO terms associated with DEGs. Nodes (yellow-to-red colored boxes) represent individual GO terms, where the intensity of red corresponds to the degree of enrichment, with red denoting the highest significance. Arrows indicate hierarchical connections between GO terms, demonstrating functional relationships or shared genes within the GO terms.


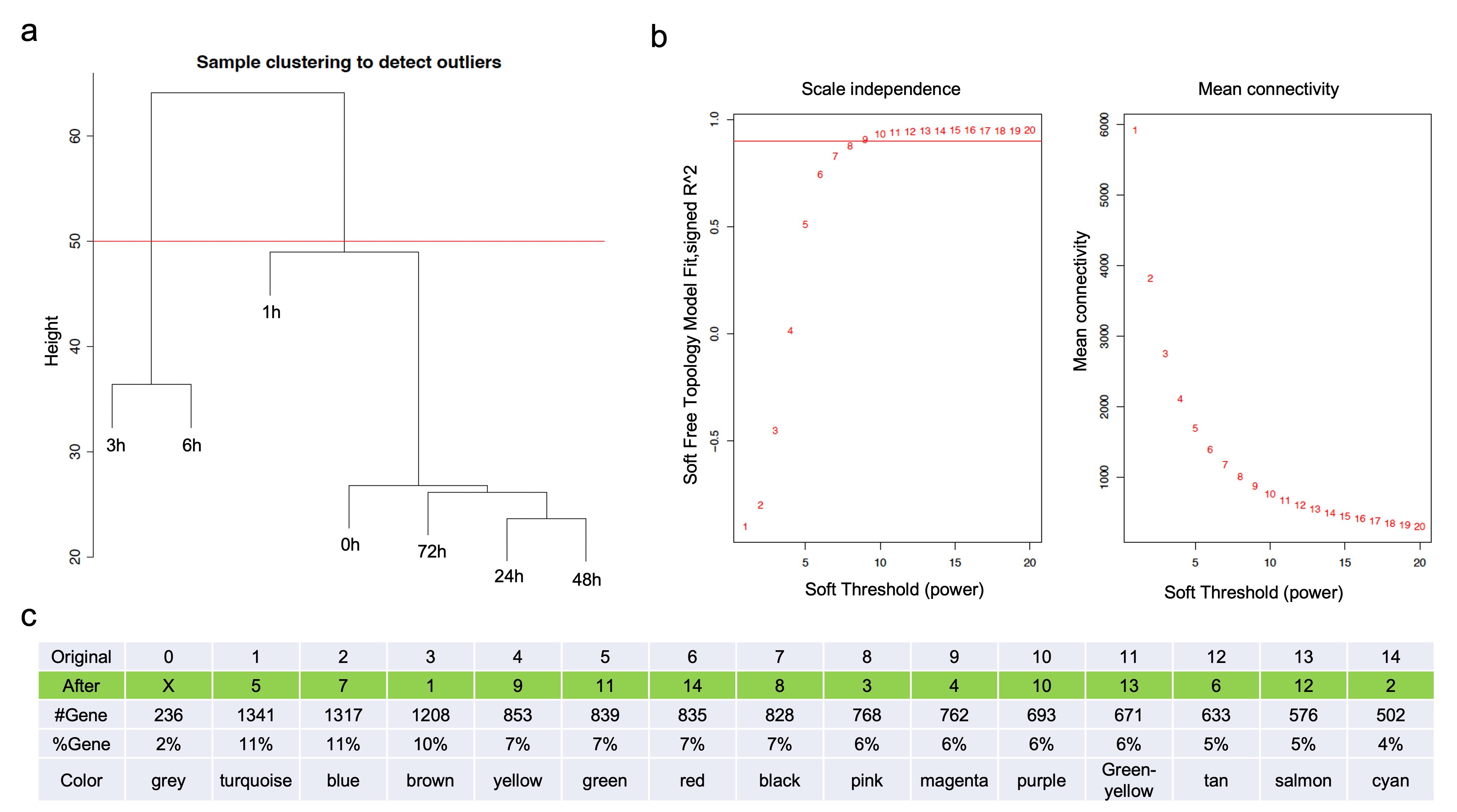


**Supplementary Figure S5.** **Determination of the soft-thresholding power in weighted gene co-expression network analysis (WGCNA).**

**(a)** Clustering dendrogram of RNA-seq samples to detect outliers based on expression profiles. Samples are clustered by similarity, and a threshold (red line) is applied to identify and exclude outliers. The clustering effectively separates samples collected at different time points (0, 1, 3, 6, 24, 48, and 72 hours) following sulfur treatment.

**(b)** Plots of network topology analysis for selecting the soft threshold power in the co-expression algorithm. The left panel shows the scale independence plot, with the y-axis representing the scale-free topology model fit (R²) and the x-axis the soft threshold power. A power of 15 (red arrow) was chosen as the optimal threshold for achieving scale-free topology. The right panel displays mean connectivity for different soft threshold powers, showing how connectivity decreases as the power increases.

**(c)** Table summarizing the results of module detection. Fourteen co-expression modules (CMs) were identified, each assigned a unique color for visualization (e.g., turquoise, blue, brown, yellow, etc.). The number of genes in each module is provided, along with the proportion (%) of genes contributing to each module relative to the total. The original module number (row name, Original) was renumbered from CM1 to CM14 (row name, After; green background) by rearranging the gene expression response time after sulfur treatment according to the co-expression pattern of the module genes.


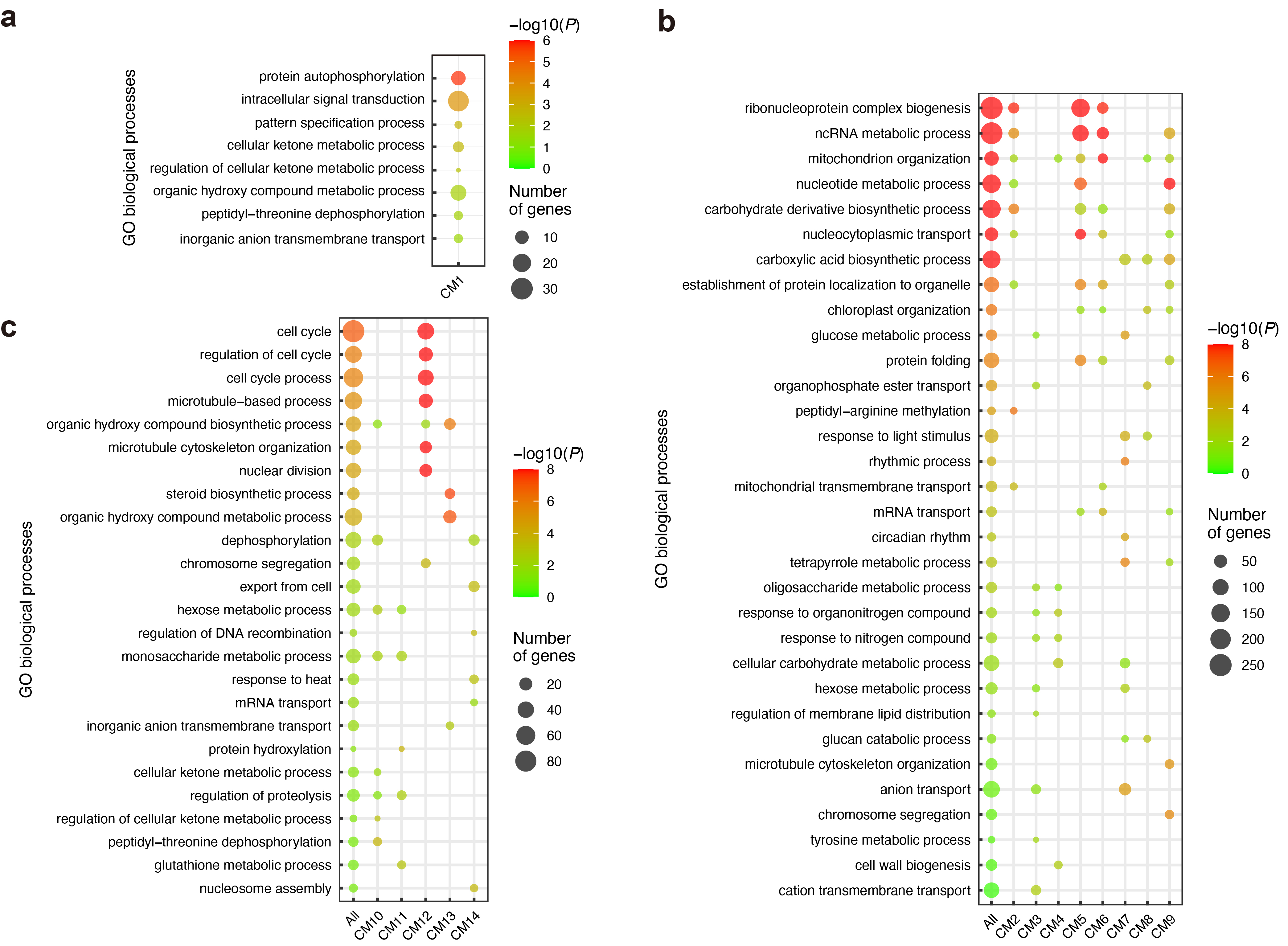


**Supplementary Figure S6. GO enrichment analysis using co-expression module genes.**

**(a)** Dot plot showing the enriched GO biological processes in module CM1.

**(b)** Dot plot displaying GO enrichment analysis for modules CM2–CM9, highlighting co-expression patterns during the early (hourly) response phase after sulfur treatment.

**(c)** Dot plot comparing enriched biological processes across modules CM10–CM14, representing co-expressed gene groups involved in the late (daily) response phase after sulfur treatment.

Dot size represents the number of genes, and color (green to red) indicates significance (-log10(P)). Biological Process (BP) GO terms with a P-value < 0.05 were selected and visualized using the ggplot2 R package.


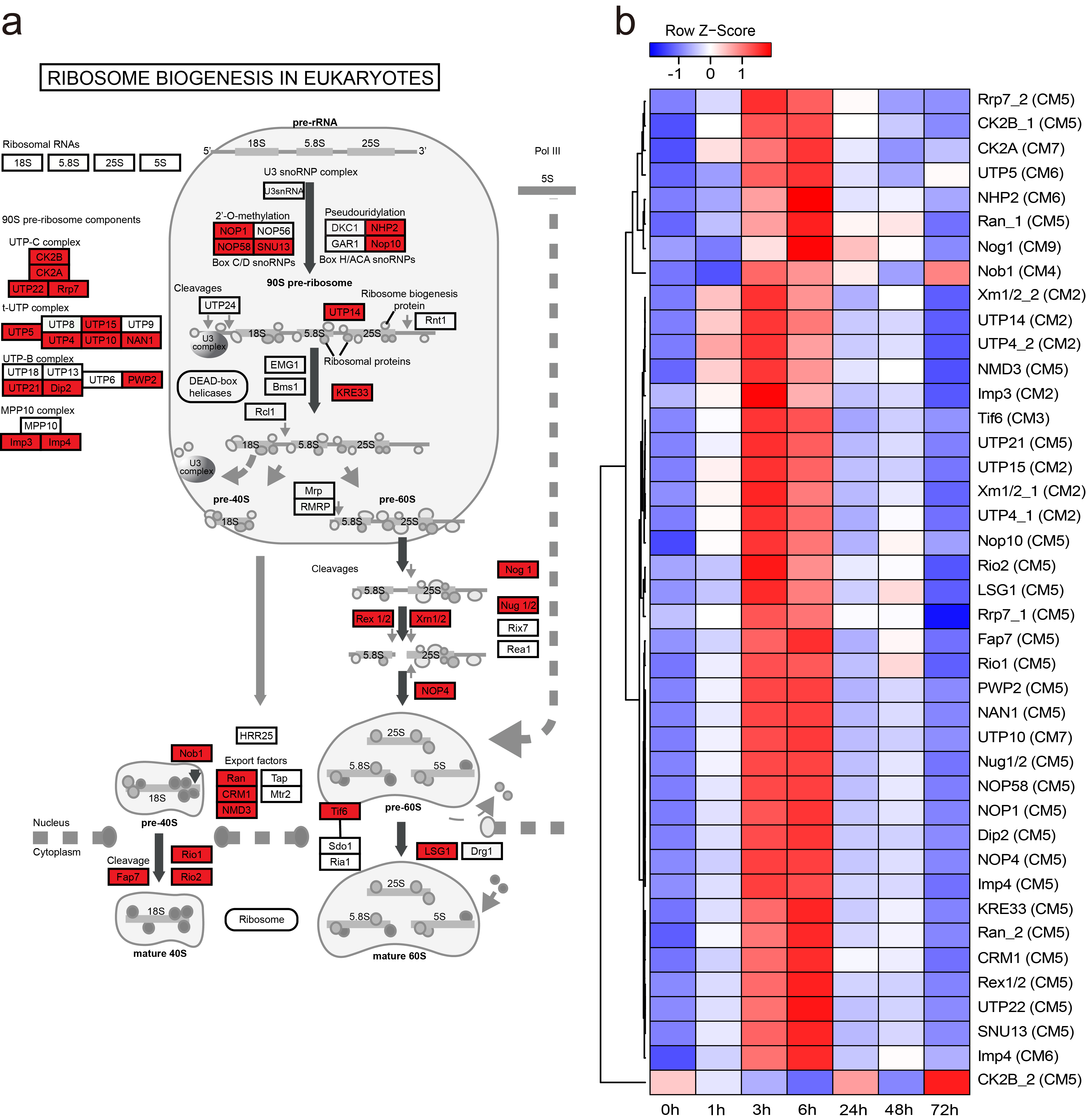


**Supplementary Figure S7. Ribosome biogenesis KEGG pathways in CM5 and CM6 with dominant gene expression changes at 3–6 hours after sulfur treatment.**

**(a)** Schematic representation of the ribosome biogenesis pathway in eukaryotes, as defined by the KEGG database. Boxes highlighted in red represent genes within CM5 and CM6 that show significant expression changes during the 3–6 hour period following sulfur treatment. The pathway illustrates the sequential assembly of ribosomal subunits, involving components of both the nucleolus and cytoplasm.

**(b)** Heatmap depicting temporal expression patterns (row Z-score) of ribosome biogenesis-related genes in CM5 and CM6 across time points (0, 1, 3, 6, 24, 48, and 72 hours) after sulfur treatment. Red bars indicate upregulated genes, blue bars indicate downregulated genes, and white bars represent neutral expression levels. Clustering reveals pronounced expression changes during the 3–6 hour period, highlighting the early transcriptional response of ribosome biogenesis pathways to sulfur treatment.

Parentheses indicate CM number, see Supplementary Table S5 for Solyc. IDs of genes used in the heatmaps in (b).


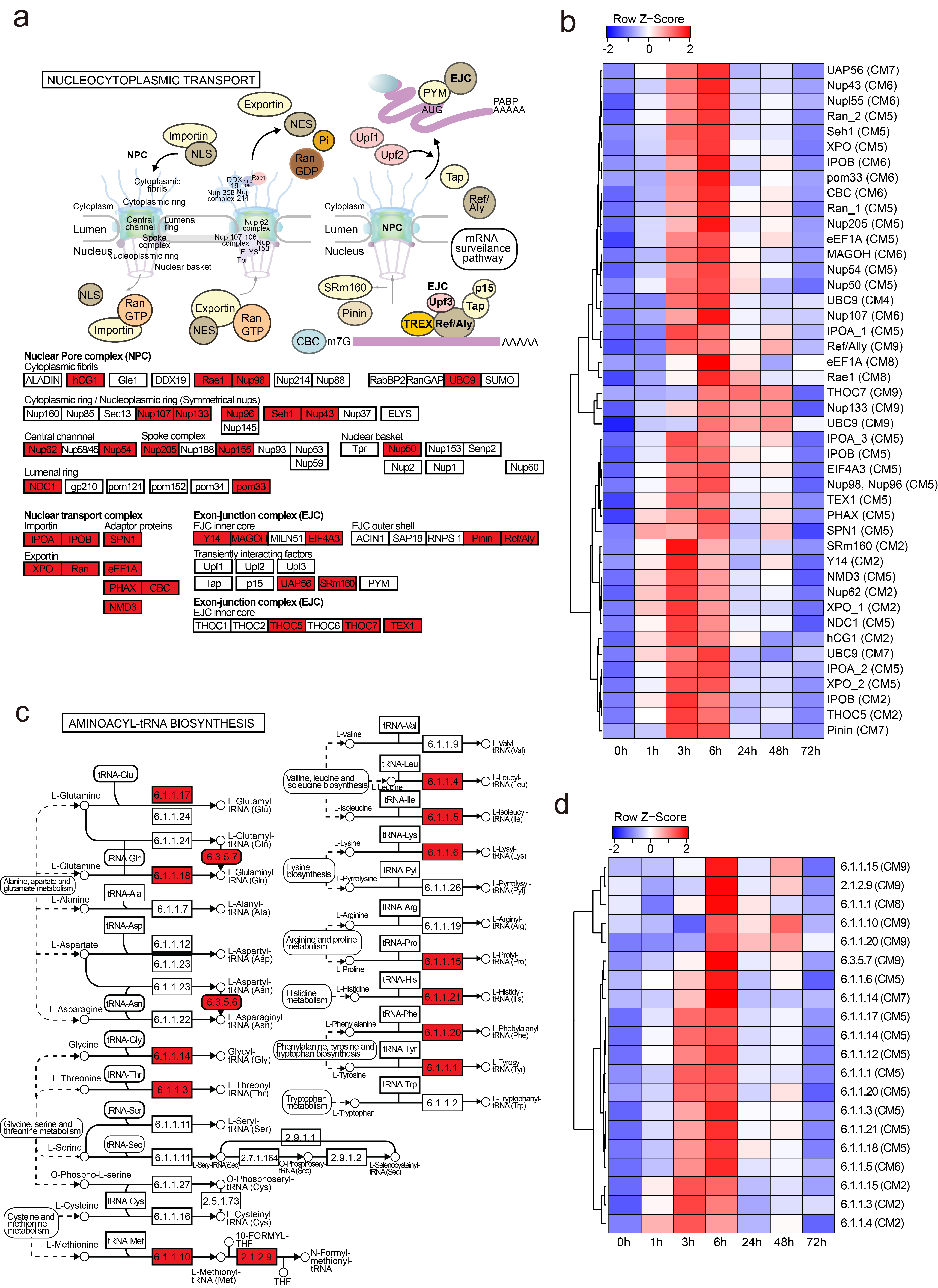


**Supplementary Figure S8. Nucleocytoplasmic transport and aminoacyl-tRNA biosynthesis KEGG pathways in CM5 and CM9 showing dominant gene expression changes after 6 hours of sulfur treatment.**

**(a, c)** Schematics of the nucleocytoplasmic transport and aminoacyl-tRNA biosynthesis pathways based on the KEGG database. Genes in CM5 and CM9 with significant expression changes at 6 hours are highlighted in red. Nucleocytoplasmic transport illustrates macromolecule movement between the nucleus and cytoplasm (a), while aminoacyl-tRNA biosynthesis highlights the attachment of amino acids to tRNAs for protein synthesis (c).

**(b, d)** Heatmaps displaying temporal expression patterns (row Z-score) of nucleocytoplasmic transport genes (b) and aminoacyl-tRNA biosynthesis genes in CM5 and CM9 across time points (0, 1, 3, 6, 24, 48, and 72 hours). Red color boxes indicate upregulated genes, blue color boxes indicate downregulated genes and white color boxes represent a neutral expression. Clustering reveals pronounced changes at 6 hours, emphasizing coordinated transcriptional responses in both pathways. Parentheses indicate CM number, see Supplementary Table S5 for Solyc. IDs of genes used in the heatmaps in (b and d).


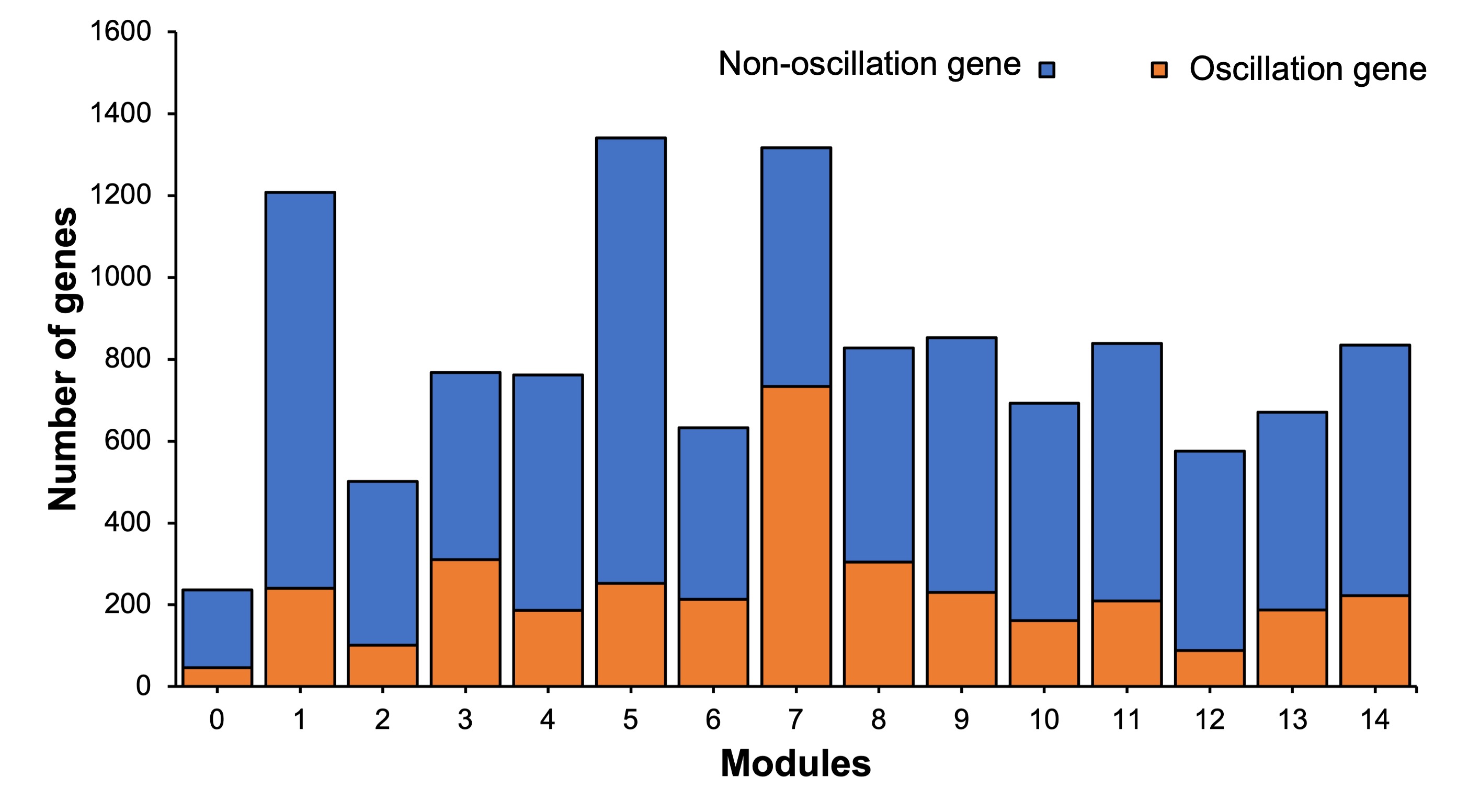


**Supplementary Figure S9. Quantification of the proportion of daily oscillation genes in co-expression module genes.**

The bar plot displays the total number of genes (blue and orange bars) and the number of daily oscillation genes (orange bars) in each of the 15 co-expressed gene modules (Modules 0–14). The y-axis represents the number of genes, while the x-axis indicates the module number.


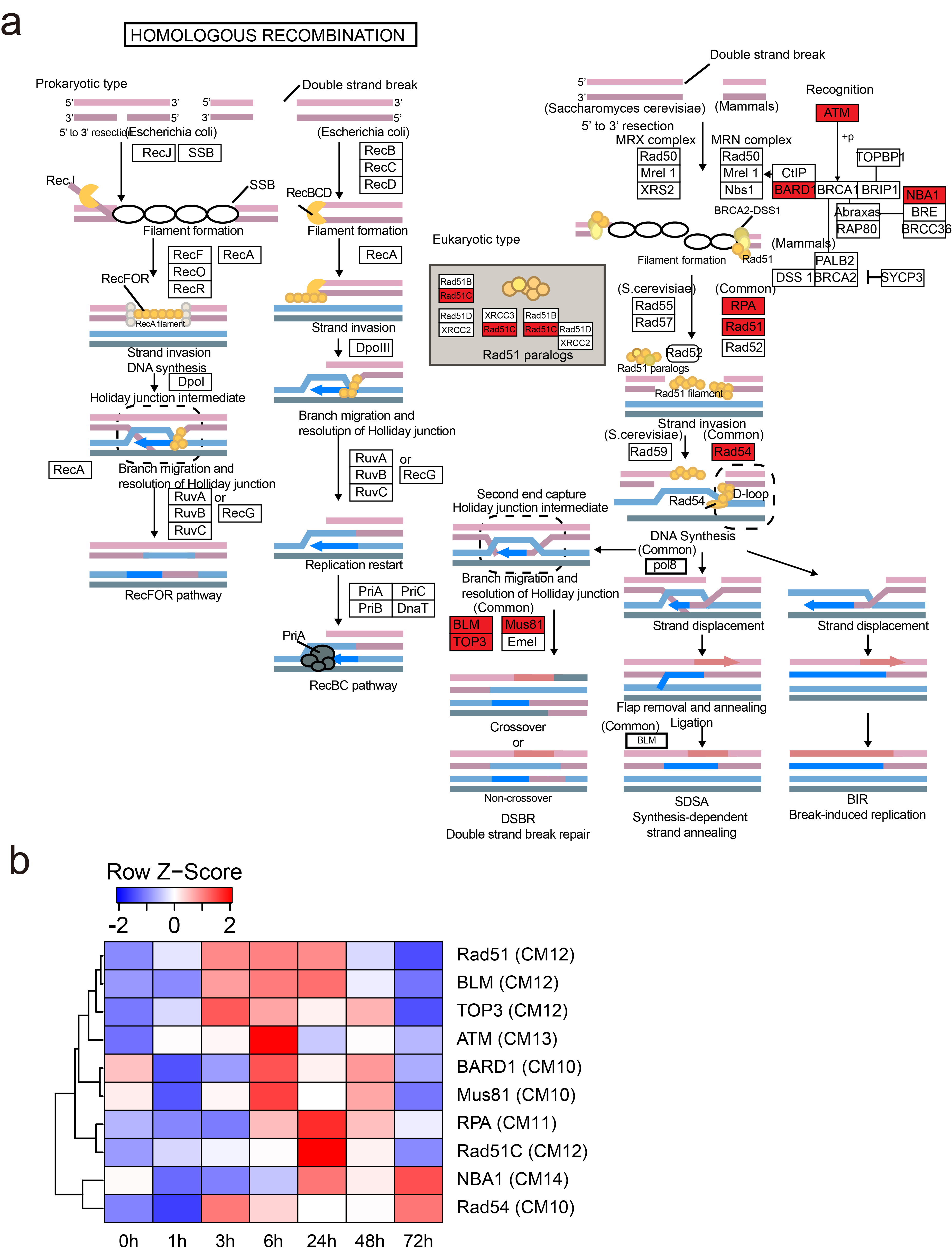


**Supplementary Figure S10. Homologous recombination KEGG pathways showing co-expression changes in daily response after sulfur treatment.**

**(a)** Schematic representation of the homologous recombination pathway in both prokaryotes and eukaryotes, as defined by the KEGG database. Genes highlighted in red belong to co-expression modules (CM10, CM12, CM13, and CM14) and exhibit significant expression changes after sulfur treatment.

**(b)** Heatmap showing temporal expression patterns (row Z-score) of homologous recombination-related genes in modules CM10, CM12, CM13, and CM14 across time points (0, 1, 3, 6, 24, 48, and 72 hours) after sulfur treatment. Red color boxes indicate upregulated genes, blue color boxes indicate downregulated genes and white color boxes represent a neutral expression. Parentheses indicate CM number, see Supplementary Table S5 for Solyc. IDs of genes used in the heatmaps in (b).

**Supplementary Figure S11.** **Criteria for Disease Severity Index (DSI) based on TYLCV symptoms.**

**(a-d)** Representative plant images showing DSI ranging from 0 to 3. 0, healthy looking. (a); 1, mild symptom (b); 2, yellowing symptom (c); 3, stunt with severe symptom (d). Scale bar, 2 cm.

**
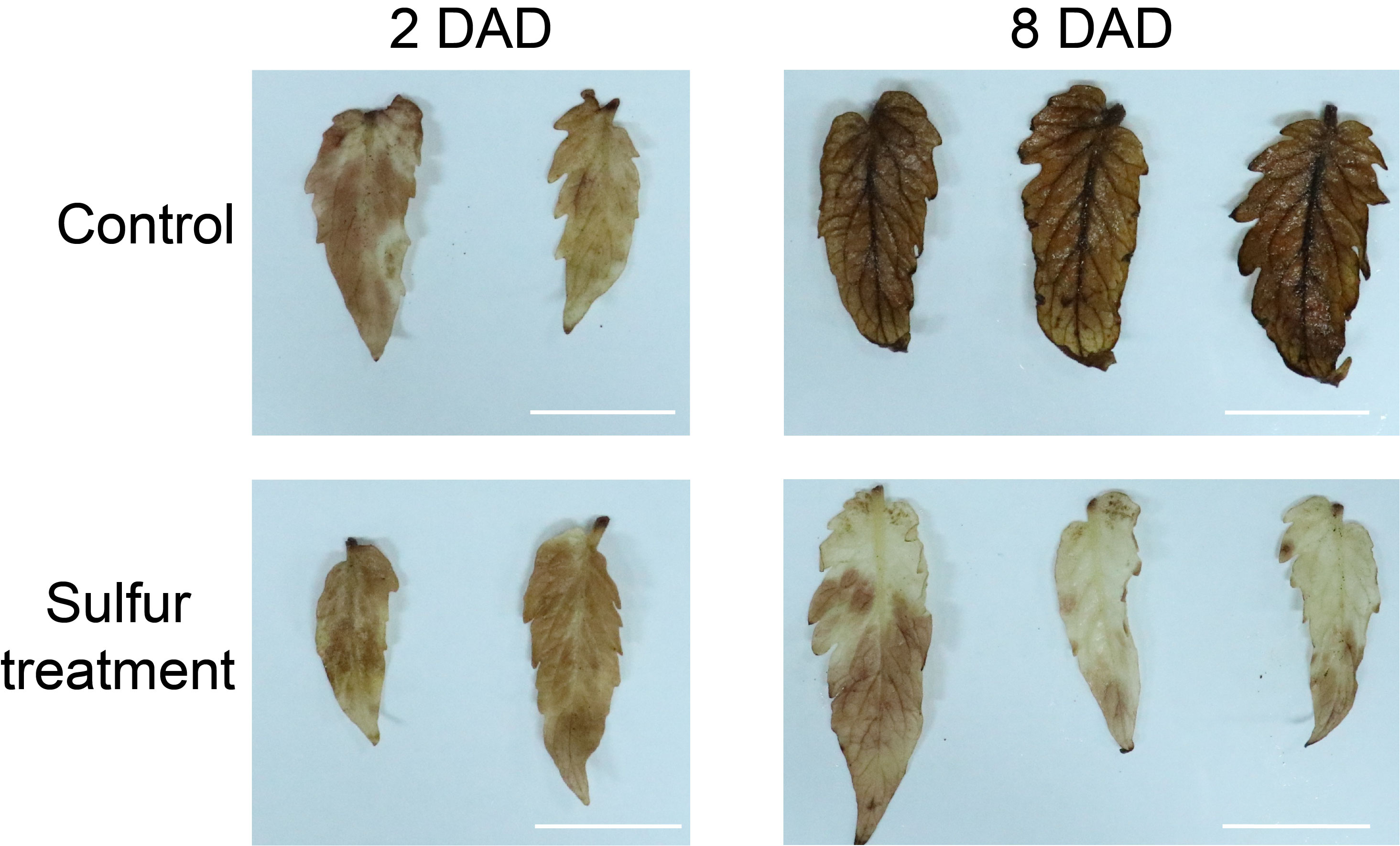
**

**Supplementary Figure S12. Reactive Oxygen Species (ROS) accumulation in leaves after drought stress.**

Representative DAB (3,3'-diaminobenzidine)-stained leaf images showing ROS accumulation in control and sulfur-treated plants at 2 and 8 days after dehydration (DAD). DAB staining was used to detect hydrogen peroxide (H₂O₂) accumulation as a marker of oxidative stress. Scale bar, 2 cm.

Elemental sulfur (25 mL of 0.4 mg/L) or distilled water (control) was applied to the soil on days 3 and 10 following the transplantation of four-week-old plants.

**
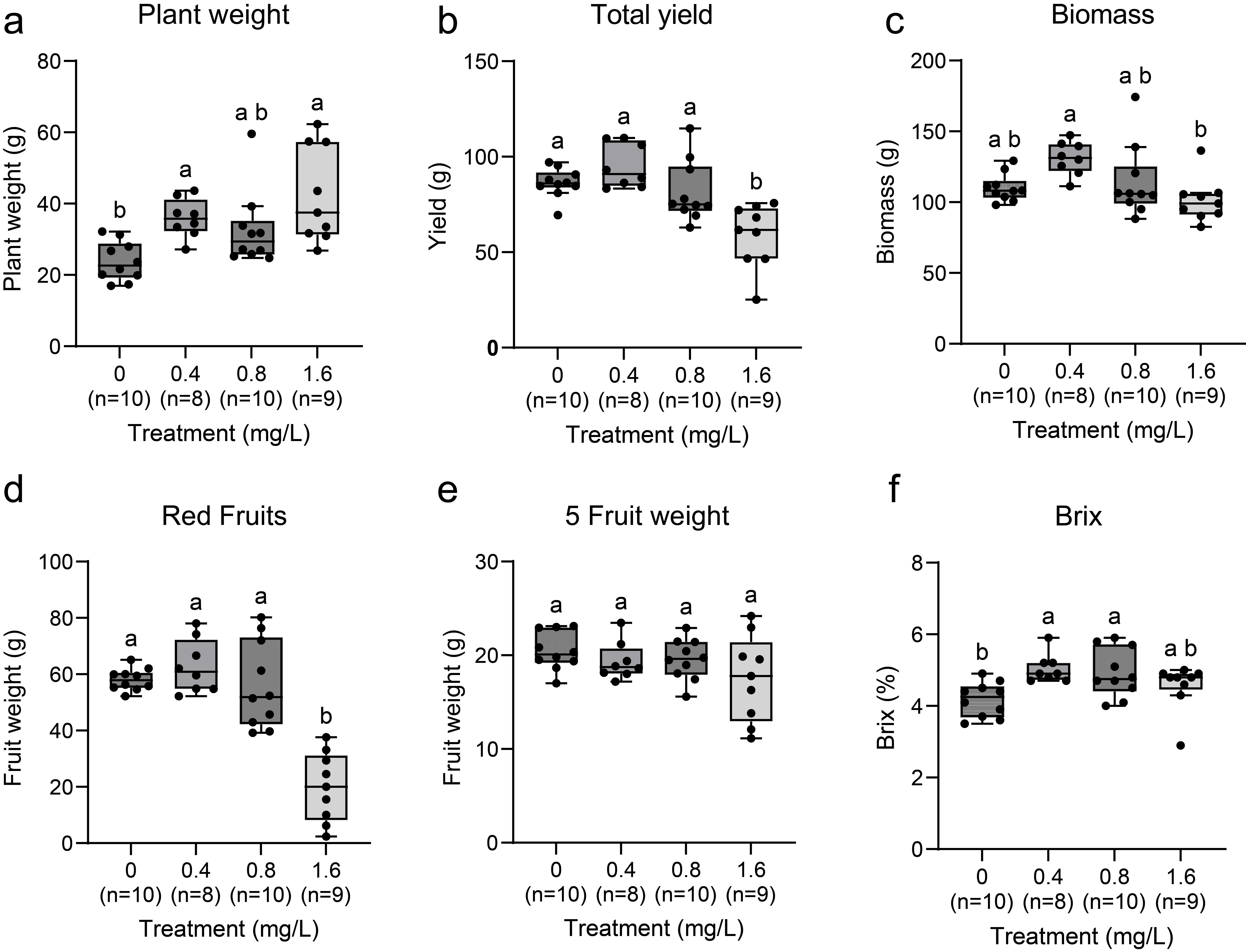
**

**Supplementary Figure S13. Quantitative comparison of tomato yield-related traits in response to fertilization with different elemental sulfur concentrations**.

**(a-f)** Box plots showing tomato yield-related traits under sulfur treatments at 0, 0.4, 0.8, and 1.6 mg/L: plant weight (a), total yield (b), biomass (c), red fruit weight (d), weight of five representative fruits (e), and Brix value (f). Statistical significance, **assessed by one-way ANOVA followed by Tukey’s HSD test, is indicated by different letters (P < 0.05). Box plots show the 25th and 75th percentiles (box boundaries), the median (bold line), and whiskers extending to 1.5× the interquartile range (IQR). Each dot represents an independent replicate. The number of plants per treatment (n) is indicated in each plot.** The 0 mg/L treatment was used as the control.

**[Original gel images used in Supplementary Figure 1a]**

Supplementary_figure_SF1a_original_1


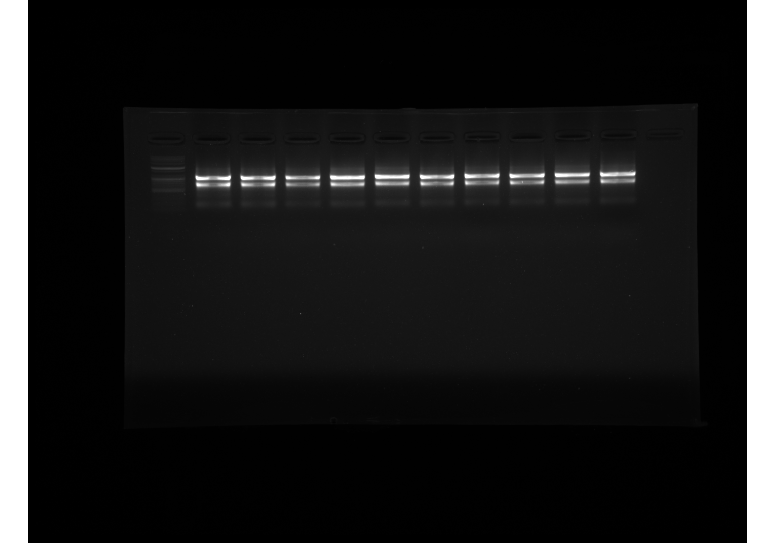


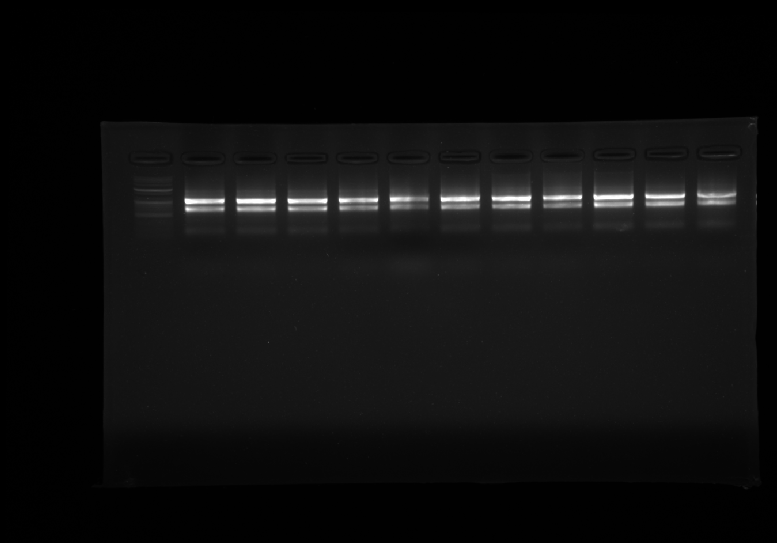
Supplementary_figure_SF1a_original_2
